# Lipid Exchangers: Cellular Functions and Mechanistic Links With Phosphoinositide Metabolism

**DOI:** 10.3389/fcell.2020.00663

**Published:** 2020-07-21

**Authors:** Nicolas-Frédéric Lipp, Souade Ikhlef, Julie Milanini, Guillaume Drin

**Affiliations:** Institut de Pharmacologie Moléculaire et Cellulaire, CNRS, Université Côte d’Azur, Valbonne, France

**Keywords:** lipid transfer proteins, lipid exchange, phosphoinositides, sterol, phosphatidylserine, membrane contact sites, signaling, vesicular trafficking

## Abstract

Lipids are amphiphilic molecules that self-assemble to form biological membranes. Thousands of lipid species coexist in the cell and, once combined, define organelle identity. Due to recent progress in lipidomic analysis, we now know how lipid composition is finely tuned in different subcellular regions. Along with lipid synthesis, remodeling and flip-flop, lipid transfer is one of the active processes that regulates this intracellular lipid distribution. It is mediated by Lipid Transfer Proteins (LTPs) that precisely move certain lipid species across the cytosol and between the organelles. A particular subset of LTPs from three families (Sec14, PITP, OSBP/ORP/Osh) act as lipid exchangers. A striking feature of these exchangers is that they use phosphatidylinositol or phosphoinositides (PIPs) as a lipid ligand and thereby have specific links with PIP metabolism and are thus able to both control the lipid composition of cellular membranes and their signaling capacity. As a result, they play pivotal roles in cellular processes such as vesicular trafficking and signal transduction at the plasma membrane. Recent data have shown that some PIPs are used as energy by lipid exchangers to generate lipid gradients between organelles. Here we describe the importance of lipid counter-exchange in the cell, its structural basis, and presumed links with pathologies.

## Introduction

Most units of life can be described as a lipidic membrane that encloses an internal and aqueous compartment hosting replication and division machinery, illustrating the fundamental role of lipidic membranes in life. Cells are of course much more complex, with internal membrane-delimited organelles that compartmentalize biochemical reactions. These membranes, with a bilayer structure, result from the assembly of a myriad of lipids together with proteins. The lipid chemistry is diverse with thousands of subspecies. All of these are precisely distributed in the cell. A nanometric snapshot of a cell would reveal that the membrane of the endoplasmic reticulum (ER), the nuclear envelope and the *cis*-side of Golgi stacks mostly contain phosphatidylcholine (PC), a neutral glycerophospholipid, phosphatidylethanolamine (PE) and phosphatidylinositol (PI), with disordered acyl chains. This snapshot would indicate that, contrastingly, the *trans*-Golgi, endosomes and the plasma membrane (PM) contain phospholipids whose acyl chains are much more ordered but also more phosphatidylserine (PS), which is an anionic lipid. Moreover, these membranes contain an important amount of sphingolipid and sterol, which is a rigid lipid (Drin, 2014).

Consequently, organelle membranes differ in term of thickness, fluidity, and their surfaces display distinct electrostatic properties in addition to hosting specific signaling capacity. Thereby each organelle membrane offers an environment that is perfectly suited for the activity of particular subsets of integral membrane and peripheral proteins. Thus, the lipid distribution observed inside the cell supports functions as diverse as endocytosis and exocytosis, signaling pathways, ionic exchange, cellular movement, respiratory function, etc.

Many processes continuously mix membranes (for example vesicular trafficking), or consume lipids (for example signaling cascades), and thereby alter the intracellular lipid distribution. To counteract this, mechanisms that create and maintain the lipid content of organelles are constantly in action. Enzymatic metabolic pathways ensure the synthesis, interconversion and degradation of lipid subspecies. In parallel, different mechanisms transfer lipids between and within the cell membranes.

Early on, it has been suspected that transfer routes were at the core of the lipid distribution because most of the lipids or lipid precursors are made in the ER. Therefore, mechanisms are required to export lipids across the cytosol toward the Golgi complex, the PM or mitochondria. Due to the hydrophobic nature of lipids, this should take hours or even days to occur spontaneously, a timescale that is utterly incompatible with cellular functions. Today it is widely assumed that the cell largely relies on Lipid Transfer Proteins (LTPs) that contain a hydrophobic cavity to shield lipids from water and catalyze lipid transfer between organelles (Lev, 2010; Wong et al., 2017). It is equally appreciated that these transfer processes partially take place in membrane contact sites (MCSs), i.e., zones of close apposition (<30 nm) between the ER and the PM or the ER and other organelles (for a recent review, see Prinz et al., 2020).

**FIGURE 1:**
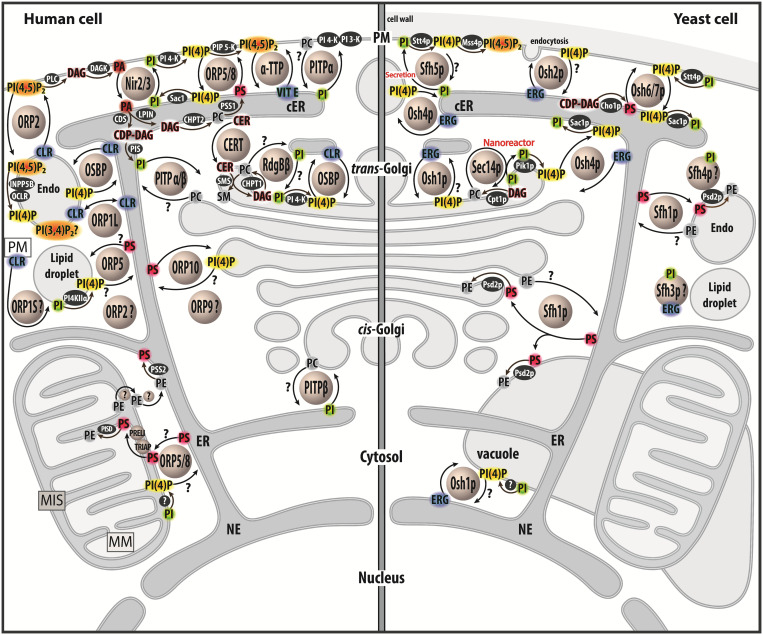
Map of lipid exchange mechanisms coupled to PIP metabolism in eukaryotes. This map indicates the localization of LTPs, which belong to the Sec14, PITP or OSBP/ORP/Osh family, and are mostly lipid exchangers in human and yeast cells. It indicates how PIP metabolism is spatially organized in the cell and is associated with PI or PIPs-related exchange processes. LTPs are represented by circles. Enzymes of lipid metabolic pathways are colored in black. An inset symbolizes the PM close to the lipid droplet. LTPs with poorly defined localization/function are labeled with a question mark. An arrow with a question mark corresponds to a lipid transfer process whose occurrence is unverified. A lipid with a question mark indicates that its presence in an organelle remains hypothetical. Circles with a question mark: unknown protein. PM, plasma membrae; NE, nuclear envelope; ER, endoplasmic reticulum; cER, cortical ER; endo, endosome; MIS, mitochondrial intermembrane space; MM, mitochondrial matrix; PC, phosphatidylcholine; PS, phosphatidylserine; PE, phosphatidylethanolamine; ERG, ergosterol; CLR, cholesterol; CER, ceramide; SM, sphingomyelin; DAG, diacylglycerol; CDP-DAG, cytidine diphosphate-DAG; PI, phosphatidylinositol; PI(4)P, phosphatidylinositol 4-phosphate; PI(4,5)P_2_, phosphatidylinositol 4,5-bisphosphate; PI(3,4)P_2_, phosphatidylinositol 3,4-bisphosphate; VIT E, vitamin E.

These LTPs belong to diverse families and show a great structural diversity (Chiapparino et al., 2016). One can distinguish several types of LTPs: those that capture only one lipid ligand, host a few lipids or that accommodate two different lipid ligands. This review will focus on this third class of LTPs, showing that most of these execute heterotypic lipid exchange between two organelles. Strikingly, a shared feature of all of these exchangers is the use of PI or phosphoinositides (PIPs) as lipid ligand (Figure 1). PI accounts for ∼10% of cellular glycerophospholipids (Vance, 2015) and consists of a glycerol backbone that bears two hydrophobic acyl chains and an inositol ring as the polar head. Importantly, PI is the precursor for a group of seven phosphoinositides (PIPs) that act as key signaling lipids. They are generated by PIP kinases and phosphatases that add or remove phosphate groups to a specific (3-, 4-, or 5-) position of the inositol ring. PIPs are produced in little quantities (less than 1% of total glycerophospholipids) in a tightly controlled manner (Di Paolo and De Camilli, 2006; Sasaki et al., 2009). Consequently, organelles harbor trace amounts of specific PIPs, which constitute molecular signposts and support various functions: signaling pathways, vesicular trafficking, cytoskeletal dynamics and ion transport. Notably, phosphatidylinositol 4-phosphate (PI(4)P) is present in the *trans*-Golgi and the PM, whereas phosphatidylinositol 4,5-bisphosphate (PI(4,5)P_2_) is restricted to the PM. Herein, we describe the tight links between PI/PIP metabolism and lipid exchangers that belong to three families, namely the Sec14p, PITP and OSBP/ORP/Osh families. We will show that these connections impart the LTPs with a unique and central role in the cell, at the interface between lipid metabolism, cellular signaling and vesicular trafficking.

## PC/PI Exchange by Sec14p and the Nanoreactor Model

PI(4)P labels the cytosolic leaflet of the *trans*-Golgi where it plays an essential role in vesicular trafficking. For instance, in yeast, PI(4)P, along with the small G protein Arf1p, recruits tetrameric adaptor proteins during the formation of vesicles that supply endosomal and vacuolar compartments with cargo proteins (Conibear and Stevens, 1998) and Gga2p (Demmel et al., 2008) to generate dense secretory vesicles for invertase secretion (Harsay and Schekman, 2002). PI(4)P is also critical in polarized exocytosis, i.e., the biogenesis of secretory vesicles that supply the PM with lipids and proteins during asymmetric cell division. PI(4)P cooperates with the small G protein Ypt32p to initiate a process that involves several downstream proteins, including Sec2p, Sec4p, and Myo2p (Ortiz et al., 2002; Santiago-tirado et al., 2011). Inactivating Pik1p, the unique PI 4-kinase that synthesizes PI(4)P at the Golgi complex, blocks almost all transport routes (Walch-Solimena and Novick, 1999).

Seminal studies in the late eighties showed that Sec14p, a cytosolic protein of ∼ 35 kDa, and the prototypical member of the CRAL-Trio superfamily, is essential to regulate Golgi PI(4)P levels and the secretory competence of the yeast. A key observation is that deleting Sec14p is lethal in yeast, vesicles fail to bud from the Golgi complex, and proteins accumulate within this organelle (Bankaitis et al., 1989, 1990). Intriguingly Sec14p can transfer PI and PC between membranes, unveiling a novel link between lipid transport and vesicular trafficking (Aitken et al., 1990; Bankaitis et al., 1990). Silencing Sac1p, which is ER-localized and the main PI(4)P phosphatase in yeast, bypasses the requirement of Sec14p for cellular viability (Whitters et al., 1993; Rivas et al., 1999). PI(4)P is not hydrolyzed into PI and becomes overabundant, counterbalancing a low PI(4)P production arising from the lack of Sec14p. Accordingly, when Sec14p is absent, the amount of cellular PI(4)P is reduced by half (Schaaf et al., 2008) and the level of available PI(4)P drops at the Golgi surface (Fairn et al., 2007). This resembles what is seen when Pik1p is missing and can be corrected by overexpressing this protein (Hama et al., 1999). Thus, Sec14p and Pik1p cooperate to generate a Golgi PI(4)P pool that can be downregulated by Sac1p.

Intriguingly, the disruption of PC production in Golgi also bypasses the requirement of Sec14p for yeast viability, suggesting that Sec14p modulates other facets of lipid homeostasis that are important for secretion (Cleves et al., 1991; McGee et al., 1994). Sec14p was found to be a repressor of the CDP-choline pathway, one of the two pathways for PC biosynthesis. When absent, PC is generated from diacylglycerol (DAG), resulting in a DAG overconsumption. DAG is critical for vesicle biogenesis, albeit in minor quantities, due to its conical shape and signaling capacity (Baron and Malhotra, 2002; Shemesh et al., 2003; Inés Fernández-Ulibarri et al., 2007; Sophie Mokas et al., 2009; Cruz-Garcia et al., 2013). At high concentrations, DAG promotes vesicle fission by locally creating regions of negative curvature (Shemesh et al., 2003). Thus, the function of Sec14p would be to tune the Golgi DAG and PI(4)P levels to maintain a lipid composition that is permissive for vesicle biogenesis. The crystal structures of apo-Sec14p (Sha et al., 1998) and its closest homolog, Shf1p, bound to either one PI or PC molecule (Schaaf et al., 2008), indicated that Sec14p traps lipids. It has a small N-terminal lobe and a larger C-terminal lobe with a longitudinal hydrophobic cavity which can host one lipid. In Shf1p, the acyl chains of PI or PC, in an extended conformation, occupy the same space in the cavity but the headgroup of each lipid is recognized by a distinct subset of residues. Comparisons between the empty Sec14p and lipid-bound Shf1p structures suggested that, once the lipid is extracted, a helical gate, initially in an open conformation, moves to cover the lipid acyl chains and close the cavity (Schaaf et al., 2008). In comparison to PC, PI is stabilized in Sec14p by more hydrogen bonds, explaining why Sec14p’s affinity for PI is 7–16 times higher than for PC (Panagabko et al., 2003, 2019).

**FIGURE 2:**
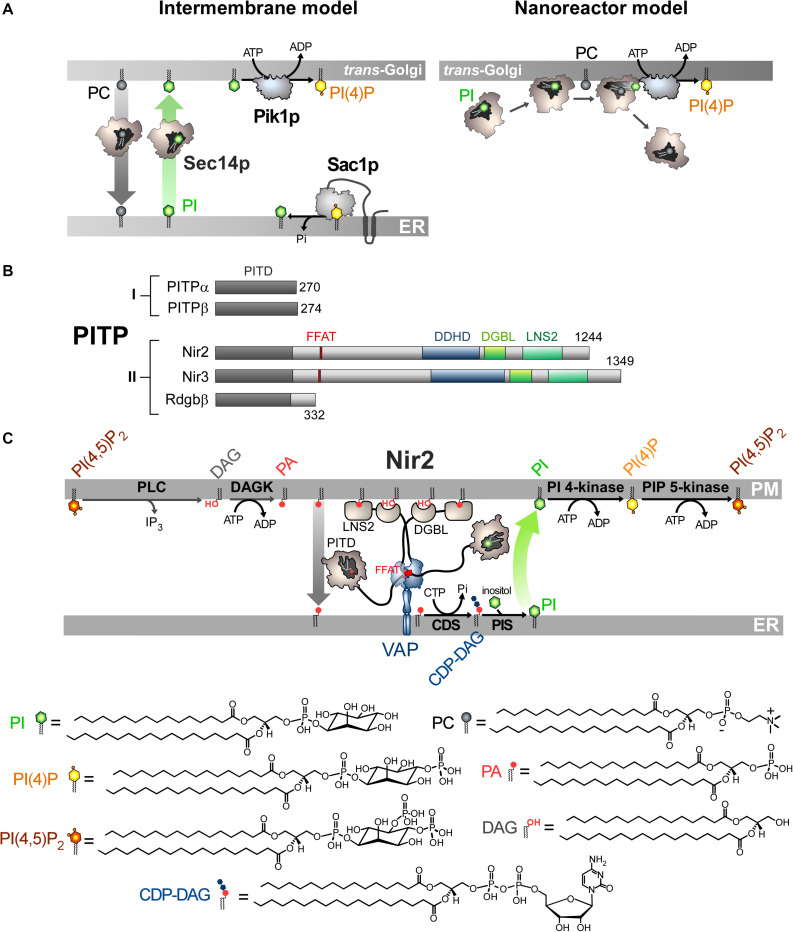
Mode of action of Sec14p and Nir2 **(A)**. *In vitro* assays indicate that Sec14p can exchange PC and PI between artificial membranes, which might suggest that Sec14p transfers PI from the ER, where it is synthesized, to the Golgi membrane. Pik1p then converts PI into PI(4)P in an ATP-dependant manner. Yet evidence rather suggests that Sec14p directly presents PI to Pik1p (nanoreactor model). The entry of PC into PI-loaded Sec14p would promote the egress of PI and its presentation to Pik1p in a configuration that is proficient for phosphorylation. **(B)** Domain organization of PI Transfer Proteins (PITP). The length of proteins in amino-acid is indicated. FFAT, two phenylalanines in an acidic tract; LNS2, Lipin/Ned1/Smp2 domain; DGBL, DAG-binding-like domain. **(C)** Nir2 associates with the ER-resident VAP protein via its FFAT motif and docks onto the PM, probably by using two domains: a LNS2 domain that recognizes PA and a DGBL domain that possibly interacts with DAG. Using a Class II PITP domain, Nir2 would execute PA/PI exchange to promote the synthesis of PI and the replenishment of the PM PI(4,5)P_2_ pool, thereby maintaining the signaling competence of the cell. PLC, phospholipase C; DAGK, DAG kinase; CDS, CDP-DAG synthase; PIS, PI synthase. The chemical structures of the different lipids that are represented in the Figure are shown.

It remains unclear how Sec14p translates its capacity to handle PC and PI into a biological function. Early investigations, using fluorescence- and radioactivity-based assays, showed that Sec14p transfers PC or PI from one membrane to another (Szolderits et al., 1989; Aitken et al., 1990). A recent approach formally established that Sec14p counter exchanges these lipids between two membranes (Sugiura et al., 2019). It is unclear whether this process offers kinetic advantages, i.e., a faster equilibration for each ligand between membranes. That said, the inclusion of PI in membranes was found to accelerate the delivery of PC preloaded in Sec14p (Panagabko et al., 2019), but it is unknown whether the opposite is true. One might suggest, based on these *in vitro* data, that Sec14p ferries PI from the ER, where it is made, to the Golgi complex to enhance PI(4)P production. Sec14p would remove PC from the Golgi complex to alleviate the toxic effect of this lipid on trafficking (Figure 2A; Cleves et al., 1991).

Many clues suggest that the intermembrane transfer activity measured *in vitro* corresponds to Sec14p activity that only occurs when given no other context or task that are available in the cell, and does not accurately reflect its cellular function. It is almost certain that Sec14p must have the dual ability to recognize PC and PI in order to fulfill its function (Schaaf et al., 2008), despite initial data (Phillips et al., 1999). A concept referred to as the nanoreactor model posits that Sec14p performs PC/PI exchange on the Golgi membrane to directly boost Pik1p activity (Schaaf et al., 2008; Bankaitis et al., 2012; Mousley et al., 2012). During a round of exchange, PC causes the egress of PI from the Sec14p cavity to form a Sec14p intermediate presenting the PI headgroup in a configuration that is competent for phosphorylation by Pik1p (Figure 2A); then Sec14p traps a new PI molecule and the PC molecule is expelled. Interestingly, the nanoreactor model assigns a dual role for Sec14p as a PC sensor and PI-presenting device that transmits PC metabolic information to the PI(4)P factory. If the DAG pool, which is crucial for TGN trafficking, is exhausted by the CDP–choline pathway for making PC, a PC pool is created, stimulating the exchange activity of Sec14p and PI(4)P synthesis. Then, higher PI(4)P levels supersede the DAG shortage to preserve vesicular trafficking. This model is difficult to test and initial attempts showed that a PC-binding mutant of Sec14p still elicits PI 4-kinase activity in the presence of PI-rich membrane (Schaaf et al., 2008). Also, given the higher affinity of Sec14p for PI vs PC and that PI is slightly more abundant than PC in the Golgi membrane (Klemm et al., 2009), it is uncertain that Sec14p performs fast PC/PI exchange. Moreover, there is no evidence that Sec14p physically interacts with Pik1p (Bankaitis et al., 2012). Although the structure of its human homolog PI4KIIIβ is known (Burke et al., 2014), we still do not know precisely how Pik1p recognizes and phosphorylates PI.

## Sec14 Homologs in Yeast

Yeasts also express five Sfh proteins (Sec14 homologs). Sfh1p is quite similar to Sec14p whereas the Sfh2-5p are more divergent (Li et al., 2000). Sfh1p has both nuclear and cytosolic localization but it can also target endosomes, the Golgi complex and vacuoles (Schnabl et al., 2003; Mizuike et al., 2019). Sfh1p has been used as a surrogate of Sec14p for structural studies and has been crystallized with PC or PI (Schaaf et al., 2008). However, overexpressing Sfh1p does not fully restore Sec14p function in yeast and barely transfers PI or PC *in vitro* (Li et al., 2000; Mizuike et al., 2019; Panagabko et al., 2019). Interestingly, single amino-acid substitutions confer Sfh1p with the ability to substitute for Sec14p, by changing the rate at which PC and PI cycles in and out the binding pocket (Schaaf et al., 2011). Sfh1p traps other phospholipids, PS and PE, and it can be crystallized with PE (Schaaf et al., 2008). Moreover, recent findings suggest that Sfh1p transports PS in yeast to supply PS from the ER to Psd2p, which decarboxylates PS into PE at the endosome surface (Mizuike et al., 2019). It has been suggested that Sfh1p acts as a PS/PE exchanger and delivers PE to the ER.

Like Sfh1p, the other Sfh proteins have PI but not PC transfer activity (Li et al., 2000). Despite this and their low sequence identity with Sec14p, Sfh2p, and Sfh4p can replace Sec14p when they are overexpressed (Li et al., 2000). *In vitro*, Sfh2p is able to transfer squalene but how this relates to its activity is unclear (Tripathi et al., 2019). Sfh4p functions in ER-endosome contact sites to activate the PS-to-PE conversion by Psd2p. Sfh4p interacts physically with Psd2p and is regulated by PI(4)P. It is unclear how PS is transferred to endosomes (Sfh4p does not transfer PS) and why Sfh4p has PI binding ability (Riekhof et al., 2014; Wang et al., 2020).

Sfh3p has been co-crystallized with PI (Yang et al., 2013) and can alternately encapsulate and transfer sterol (Holič et al., 2014; Tripathi et al., 2019). Sfh3p is linked to ergosterol metabolism and controls a PI(4)P-dependent signaling pathway that regulates the use of lipids at lipid droplets (LDs), which are energy-storage organelles, full of triacylglycerol (TAG) and steryl-ester and covered by a lipid monolayer, that originate from the ER (Holič et al., 2014; Ren et al., 2014). However, it is unclear exactly how Sfh3p functions. Sfh5p is involved in polarized exocytosis by supplying PI to secretory vesicles that fuse with the PM. PI is sequentially converted into PI(4)P and PI(4,5)P_2_ by the PI 4-kinase, Stt4p, and the PIP 5-kinase, Mss4p. PI(4,5)P_2_, with the small G protein Cdc42p, govern the organization of the actin skeleton at the PM that assists polarized exocytosis (Liat and Gerst, 2009). Recent computational approaches indicated that Sfh proteins have evolved differently and each contain a specific cavity microenvironment while conserving a common “barcode,” i.e., a few residues at specific positions in the sequence to recognize PI (Tripathi et al., 2019). This gives them specific cellular functions with or even without links to PI/PIP metabolism.

## Mammalian Sec14-Like Proteins Capture and Transfer Lipophilic Ligands

α-TTP (α-tocopherol transfer protein) is a Sec14p-like protein with high specificity for α-tocopherol (Panagabko et al., 2003), the most abundant form of vitamin E in mammals, to ensure its secretion from liver cells. Autosomal recessive mutations in α-TTP provoke strong neurological disorders (e.g., ataxia) linked to a deficiency in circulating vitamin E (Mariotti et al., 2004). Intriguingly, a mutation that provokes a severe, early-onset form of the disease does not prevent α-TTP from sequestering α-tocopherol. Instead, it decreases its capacity to recognize PI(4,5)P_2_ or PI(3,4)P_2_. In fact, these PIPs guarantee the efficient transfer of α-tocopherol to the PM. *In vitro*, α-TTP exchanges α-tocopherol for PI(4,5)P_2_ between membranes (Kono et al., 2013) demonstrating that α-TTP is a vitamin E/PIP exchanger in liver cells.

CRALBP (cellular retinaldehyde-binding protein) is a key component of the visual cycle of vertebrates that binds vitamin A; its malfunctioning leads to several vision pathologies (Saari and Crabb, 2005). In photoreceptor cells, when a photon hits opsin, it induces the photoisomerization of 11-*cis*-retinal (11-*cis*-RAL) to all-*trans*-retinal (all-*trans*-RAL) linked to that receptor. All-*trans*-RAL is reduced into all-*trans*-retinol and transferred to retinal pigment epithelium (RPE) cells. All-*trans*-retinol is subsequently esterified and the all-*trans*-retinyl ester is converted into 11-*cis*-retinol by an isomerohydrolase. CRALBP enhances this process, likely by preventing the inhibition of the enzyme by the end-product of the reaction. CRALP also serves as a substrate carrier for 11-*cis*-RDH (RDH5), facilitating the oxidation of 11-*cis*-retinol to 11-*cis*-RAL (Saari et al., 1994; Winston and Rando, 1998; Stecher et al., 1999). CRALBP then delivers 11-*cis*-RAL to the PM of RPE for export to the adjacent photoreceptor cells. CRALBP has a high affinity for 11-*cis*-RAL but cannot bind lipids such as PC or PI (Panagabko et al., 2003). However, structural analyses indicate that the mode of association of CRABLP with its ligand resembles that which is observed for the lipid-bound Sfh1p (He et al., 2009). The CRALBP structure also revealed that a mutation that causes Bothnia dystrophy induces a rearrangement of the Sec14-like domain, thus preventing the release of 11-*cis*-RAL from the binding pocket (He et al., 2009). Intriguingly, acidic PM phospholipids, such as PS and PA, might enhance the egress of 11-*cis*-RAL from CRALBP but probably not by an exchange process (Saari et al., 2009).

Another mammalian Sec14-like protein that is suspected to have an intracellular transfer activity is the Supernatant Protein Factor (SPF, also referred to as α-Tocopherol Associated Protein). It stimulates the conversion of squalene to lanosterol, and thus promotes cholesterol biosynthesis, presumably by transferring squalene to metabolically active specific membrane sites (Shibata et al., 2001). SPF is also linked to tocopherol metabolism (Porter, 2003). The structure of its Sec14-like domain, both empty and bound to squalene, is known (Stocker et al., 2002; Christen et al., 2015). It has some affinity for PI (Panagabko et al., 2003) but it is unknown whether this is important for its function.

## Class I PITP Are PC/PI Exchangers That Regulate Golgi Functions

In humans, five proteins constitute the PITPs family (Figure 2B; Cockcroft and Carvou, 2007). All of these contain a PI-transfer domain (PITD). Pioneer studies indicated that PITPs of Class I, referred to as PITPα and β, could transfer PC or PI between membranes, suggestive of a capacity to act as PC/PI exchangers between organelles (Helmkamp et al., 1974; Van Paridon et al., 1987; De Vries et al., 1995). Recently, it has been shown that the Class II PITPs correspond to PA/PI exchangers. As detailed below, this has a fundamental implication for phospholipase C (PLC)-based signaling pathways that generate second messengers, DAG and inositol 1,4,5-trisphosphate (IP3), by hydrolyzing PI(4,5)P_2_ upon receptor activation.

The first class of PITPs is composed of PITPα and β. PITPα is highly expressed in the brain and is predominantly localized in the axons (Cosker et al., 2008) whereas PITPβ is abundant in the liver and localizes to the Golgi complex and the ER (Morgan et al., 2004; Phillips et al., 2006; Shadan et al., 2008). These isoforms have only a PITD with the capacity to trap PI or PC and to transfer them between membranes (Van Paridon et al., 1987). The crystal structures of the rat PITPα and human PITPβ have been solved both empty and loaded with PC or PI (Schouten et al., 2002; Tilley et al., 2004; Vordtriede et al., 2005). The PITD consists of an eight-stranded β-sheet flanked by two long α-helices that form a pocket that can host either one PC or one PI molecule. An α-helix (G-helix), along with 11 C-terminal amino acid residues function as a gate to close the cavity. The polar head of the lipid is embedded inside the pocket: the inositol ring of PI makes contacts with four amino acids which are conserved in the PITD of many proteins (Schouten et al., 2002; Tilley et al., 2004; Vordtriede et al., 2005). In contrast, it is unclear how PC is recognized. The strong network of H-bonds between the PI molecule and the pocket residues explain why PITPα and β have 16-times more affinity for PI than for PC (Van Paridon et al., 1987; Tilley et al., 2004). A structural comparison of apo and lipid-loaded forms of PITP suggests that the G-helix swings out to allow the release of the lipid from the pocket. In its open and empty state, the PITD can dock onto membrane thanks to a larger hydrophobic interface (Tilley et al., 2004; Shadan et al., 2008).

The two PITPs execute distinct but redundant functions. Early data suggested that PITPα assists PLCβ- or PLCγ-based signaling cascades in permeabilized cells (Thomas et al., 1933; Kauffmann-Zeh et al., 1995). Notably, it was proposed that the EGF-receptor recruits PITPα, PLCγ and PI 4-kinase to generate a robust signal by ensuring both the genesis of PI(4,5)P_2_ and its conversion into second messengers. PITPα was reported to sustain the production of PI(3,4,5)P_3_ by PI 3-kinase in human neutrophils in response to a chemoattractant (Kular et al., 1997). Other reconstitution assays showed that PITPα promotes the formation of secretory vesicles from the *trans*-Golgi network (TGN) (Ohashi et al., 1995) or the fusion of secretory granules with the PM (Hay and Martin, 1993). These studies conveyed the idea that PITPs, and more broadly PC/PI exchangers, could assist PIP metabolism and PIP-dependent signaling given that Sec14p could substitute for PITPs in some of these assays. However, most of them did not formally demonstrate that PITPα impacts PIPs levels. Subsequent data indicated that the physiological role of PITPα is related to the development and function of the nervous system. In mice with the *vibrator* mutation, a 5-fold decrease in PITPα expression leads to tremor, degeneration of the brain stem and spinal cord neurons, and early death (Hamilton et al., 1997). Full ablation of the PITPα gene in mice results in premature death associated to cerebellum diseases, hypoglycaemia, and intestinal and hepatic steatosis due to defects in ER function in different cell types (Alb et al., 2003). Recently, PITPα has been shown to maintain the PIP pools dedicated to PLC and PI 3-kinase-dependent signaling during axonal growth in response to external growth factors, to proteins of the extracellular matrix or to netrin-1, an extracellular guidance cue (Xie et al., 2005; Cosker et al., 2008). Interestingly, PITPα might interact with the netrin receptor along with PI(5)P to maintain the availability of PI(4,5)P_2_ in the PM. As in the case for Sec14p, it is unclear whether PITPα provisions the PM with PI or directly presents PI to PI 4-kinases.

The function of PITPβ remains ill-defined. Preliminary investigations suggested that the absence of PITPβ is embryonic lethal in mice (Alb et al., 2002) but it has been recently reported that PITPβ-null mice are viable with no obvious phenotype (Xie et al., 2018). In cell culture, PITPβ samples the ER and Golgi surface within a few minutes, which suggests that it executes fast PC/PI exchange between these organelles (Shadan et al., 2008). It is accepted that PITPβ upregulates the Golgi PI(4)P level, but divergent results have been obtained. PITPβ seems to be critical for Golgi-to-ER retrograde trafficking, mediated by COPI-coated vesicles. PITPβ would deliver PI from the ER to the *cis*-Golgi for maintaining a PI(4)P pool to interface COPI vesicle formation with the binding of Golgi complex to the actin cytoskeleton (Carvou et al., 2010). In contrast, in neural stem cells (NSCs), PITPβ potentiates PI(4)P synthesis not at the *cis* but at the *trans*-Golgi and, interestingly, this function is also ensured by PITPα (Xie et al., 2018). The PI(4)P pool serves to recruit GOLPH3 and the non-conventional myosin MYO18A that interacts with F-actin and promotes Golgi-to-PM trafficking. The tensile force exerted by actin cytoskeletal proteins interacting with proteins bound to the TGN, enhances vesicular budding, the secretory capacity of the TGN and its positioning in the apical compartment of NSCs. This facilitates the apical sorting of cargo proteins and lipids and thereby optimizes Golgi responses to apical PM signaling. This is critical: a lack of both PITPα and PITPβ provokes a misalignment of NSCs in the neocortex followed by apoptotic events, preventing dorsal forebrain development.

## Class II PITPS Are PA/PI Exchangers That Maintain the Signaling Competence of the Cell

Phospholipase C-based signaling pathways rely on the hydrolysis of PI(4,5)P_2_ to produce second messengers. It has long been appreciated that mechanisms must be in place to rapidly regenerate the PM PI(4,5)P_2_ pool and preserve the signaling competence of the cell as well as other PI(4,5)P_2_-dependant processes (channel activation, endocytosis). This relies on the so-called PI cycle, a multi-step pathway that recycles DAG into PI(4,5)P_2_. A first and necessary step is the conversion of DAG into PA by a DAG kinase (Cai et al., 2009) that is supposed to be DGKε (Epand, 2017). Indeed, this is the only isoform that shows high specificity for DAG species with the same acyl chain composition found in the lipid intermediates of the PI cycle. However, DGKε is more clearly observable at the ER than at the PM, where it should function (Kobayashi et al., 2007; Decaffmeyer et al., 2008). Moreover, although DGKε is involved in PI(4,5)P_2_-dependant signaling cascades linked to cortex functions in mice, it is surprisingly not essential for their development and survival. Presumably, other DAG kinases can substitute for DGKε *in vivo* (Rodriguez De Turco et al., 2001). PA is then converted into CDP-DAG via the consumption of CTP by integral membrane proteins, CDP-DAG synthase (CDS1/2) (Lykidis et al., 1997). Thereafter, the PI synthase (PIS) conjugates inositol with CDP-DAG to make PI (Tanaka et al., 1996; Kim et al., 2011). Finally, PI undergoes sequential phosphorylation to generate PI(4,5)P_2_. For this cycle to function, as foreseen by Michell (1975), it is necessary to transfer PA from the PM to the ER and PI in the opposite direction. Indeed, CDS and PIS, the two enzymes that ensure the PA-to-PI conversion, reside at the ER, whereas PI is phosphorylated at the PM. It was unknown for many years how PA and PI lipids are transferred between these two compartments. As mentioned previously, in 1993 a study from the S. Cockcroft team suggested that the maintenance of PI(4,5)P_2_-based signaling in human cell lines was dependent on PITPα (Thomas et al., 1933). However, as this protein recognizes PI and PC, this did not indicate how PA is transferred. Later, the same group focused on human RdgBβ (*a.k.a.* PITPNC1), Nir2 (alias RdgBb1; PITPNM1), and Nir3 (RdgBb2, PITPNM2), which belong to the Class II of PITP and were less well characterized than Class I in respect to their lipid binding properties. RdgBβ only consists of an N-terminal PITD followed by a C-terminal extension of 80 amino-acids. Nir2/Nir3 are more complex with an N-terminal PITD followed by a FFAT motif (two phenylalanines in an acidic tract) to interact with the ER-resident VAP protein (Nishimura et al., 1999; Amarilio et al., 2005; Kim et al., 2013), and two domains, a DDHD domain, which possibly targets PI(4)P (Inoue et al., 2012) and a LNS2 (Lipin/Ned1/Smp2) domain, which was identified in the lipin proteins as possessing PA phosphatase activity. In Nir2, the LNS2 domain lacks a key catalytic residue, suggesting that Nir2 has no PA phosphatase activity but detects PA. An important study found that RdgBβ can trap PI or a second lipid, PA, and not PC (Garner et al., 2012). It is unclear why Class I and II PITD are selective for PC and PA, respectively. Possibly, a single cysteine and a bulky residue, which are critical for the recognition of PC, are replaced by threonine and alanine, respectively, in Class II PITD (Garner et al., 2012).

The PITD of Nir2 and its Drosophila homolog *Dm*-RdgBα also displays dual specificity for PA and PI. This has central implications. First, *Dm*-RdgBα is localized to the submicrovillar cisternae (SMC), a subcompartment of the ER adjacent to the microvillar PM of Drosophila photoreceptors cells (Vihtelic et al., 1993), so it is localized in a subcellular region that resembles ER–PM contact sites. Second, loss-of-function mutants in *Dm*-RdgBα are characterized by an abnormal conversion of light signal into electrical response. Moreover, the rhabdomeric membranes become vesiculated, leading to photoreceptor cell degeneration (hence the name RdgB for retinal degeneration type B). Photon absorption by the GPCR rhodopsin is transduced into electrical activity by G-protein-coupled PLCβ-mediated PI(4,5)P_2_ hydrolysis. Therefore, one might speculate that *Dm*-RdgBα and its human homolog are strongly involved in the maintenance of cellular signaling competence. In human cell lines exposed to growth factors, Nir2 translocates to the PM due to the formation of PA, thanks to its C-terminal LNS2 (Kim et al., 2013). Nir2 is required to maintain PM PI(4,5)P_2_ levels and the stimulation of PI(3,4,5)P_3_ production by growth factors that positively regulate the MAPK and PI 3-kinase/Akt pathways (Kim et al., 2013). Nir2 locates to the ER by associating with VAP proteins (Amarilio et al., 2005) and Balla and co-workers eventually showed that Nir2 colonizes ER-PM contact sites following PLC activation (Kim et al., 2015). Importantly, they showed that Nir2 transfers PA from the PM to the ER and connects PA with PI metabolism to maintain proper PM PI(4,5)P_2_ levels. Overall, this strongly suggested that Nir2 acts as a cellular PA/PI exchanger. They also identified a segment called DGBL (DAG-binding-like) with a sequence with some similarity to the DAG-binding C1 domain. This segment cooperates with LNS2 to anchor Nir2 to the PM by recognizing PLC-generated DAG. Once Nir2 is docked to the PM, the excess PA is loaded inside Nir2 PITD and transferred to the ER. PA is used to make PI which is subsequently conveyed to the PM for phosphorylation (Figure 2C). Concurrently, it was reported that *Dm*-RdgBα, whose molecular configuration is similar to that of Nir2, localizes between the SMC and the PM in photoreceptors cells to couple PA and PI turnover by PA/PI exchange and thus to sustain the PLC-based signal transduction of light (Yadav et al., 2015). Note that elevated cytosolic Ca^2+^ levels, following PLC activation, enhances the formation of ER-PM contact sites by the membrane-tethering factor E-Syt1 (Giordano et al., 2013), and helps Nir2 to regenerate PI(4,5)P_2_ (Chang et al., 2013). Interestingly, comparatively to Nir2, Nir3 detects lower level of PA in the PM via its LNS2 domain and has a lower PA transfer capacity. Consequently, Nir3 can sustain proper PI(4,5)P_2_ levels in resting cells whereas Nir2 is only mobilized during intense receptor activation to actively exchange lipids (Chang and Liou, 2015). Finally, Balla and co-workers noted that a truncated Nir2 mutant that lacks PITD is strongly enriched in ER-PM contact sites (Kim et al., 2015). This suggests that Nir2, by transferring PA to the ER, self-regulates its PA-dependant association to the PM.

Nir2 is detected in ER-Golgi contact sites in resting cells and is critical for maintaining proper DAG levels in the TGN and the secretory capacity of this compartment (Litvak et al., 2005; Kim et al., 2013). Nir2 also controls Golgi PI(4)P levels (Peretti et al., 2008). These observations have been interpreted with the prospect that Nir2 was a PC/PI exchanger. It is today unclear how Nir2, via its PA/PI exchange ability, regulates Golgi DAG or PI(4)P levels. Intriguingly, the Nir2 PITD, which is supposed to move throughout the cytosol in order to exchange lipids, ensures the association of Nir2 with the Golgi surface (Kim et al., 2013). Overall, how Nir2 functions in ER-Golgi contacts besides its role at the ER/PM interface remains a mystery.

## Role of Class II PITPs in Pathologies

There are tenuous links between Class II PITPs and cancer, likely due to their role in coupling lipid metabolism with cellular signaling. Nir2 enhances the transition of mammary epithelial and breast cancer cells into a more motile and invasive state, with a higher metastatic capacity. These effects are mainly mediated by the PI 3-kinase/Akt and the ERK1/2 pathways. Nir2 expression correlates with high tumor grades and poor disease outcomes in breast cancer patients. These new findings reveal important physiological roles of LTPs and their implication in human diseases (Keinan et al., 2014).

RdgBβ has a single C-terminal extension that, upon phosphorylation, associates with the 14-3-3 protein, and increases its stability (Garner et al., 2011; Halberg et al., 2016). Moreover, RdgBβ interacts via its PITD with ATRAP (Angiotensin II Type I Receptor-Associated Protein), a transmembrane protein that interacts with the angiotensin II receptor type 1 (AT1R) and triggers its internalization, shutting-down subsequent PLC activation, and thereby exerts a protective effect against AngII stimulation (Garner et al., 2011, 2012; Cockcroft and Garner, 2013). However, the role of this interaction is unclear. There is compelling evidence that RdgBβ is involved in the metastatic process of cancer cells (Png et al., 2012) and the mechanisms behind this have been unveiled (Halberg et al., 2016). RdgBβ localizes on the Golgi surface in a PI(4)P-dependent manner to recruit the small G protein Rab1, which in turn recruits MYO18A. This enhances the secretory activity of the TGN and the release of pro-invasive and pro-angiogenic factors out of the cancer cell, thereby driving metastasis (Halberg et al., 2016).

## A PI(4)P Concentration Gradient to Fuel Sterol Transport

The concept of lipid exchange has been greatly expanded through the characterization of oxysterol-binding protein (OSBP) and its homologs in eukaryotes. OSBP was initially identified as a cytosolic protein that bound hydroxysterols (Kandutsch and Thompson, 1980). Moreover, some clues suggested that the downregulation of sterol synthesis by hydroxysterols, most particularly 25-hydroxycholesterol (25-HC), was dependant on OSBP. At the end of the ‘80s, the cDNAs of the rabbit and human OSBP were cloned (Dawson et al., 1989; Levanon et al., 1990), allowing further molecular and cellular analyses. In 1992, Ridgway and co-workers found that the C-terminal half of OSBP contains a oxysterol-binding domain (Ridgway et al., 1992) and that OSBP relocates to the Golgi apparatus in the presence of 25-HC. This translocation depends on an N-terminal pleckstrin-homology (PH) domain of ∼ 90 aa (Haslam et al., 1993; Gibson et al., 1994; Lagace et al., 1997) that recognizes both PI(4)P and the small G protein Arf1 (Levine and Munro, 2002; Godi et al., 2004). OSBP was also found to locate to the ER by interacting with VAP (Wyles et al., 2002), thanks to an FFAT motif (Loewen et al., 2003).

The advent of genomics in the ‘90s led to the discovery of many OSBP homologs in eukaryotes. Based on sequence similarity to the ligand-binding domain of OSBP (OSBP-Related Domain or ORD) (Jiang et al., 1994; Beh et al., 2001), seven genes were identified in *S. cerevisiae* that encoded proteins specifically named Osh (Oxysterol-binding protein homologs). They were classified into four subfamilies: Osh1/2p, Osh3p, Osh4/5p (a.k.a., Kes1p and Hes1p), and Osh6/7p, on the basis of their overall sequence homology. The first three are complex with a PH domain and an ORD. Osh1p and Osh2p also contain an ankyrin repeats (AR) domain. The other Osh proteins only correspond to an ORD.

Furthermore, 11 human OSBP-related proteins (ORPs) were found, and together with OSBP, they define the ORP family (Lehto et al., 2001). They were classified into six subfamilies on the basis of sequence similarity and gene structure. Most of them resemble OSBP with a PH domain near the N-terminal end, an FFAT motif and a C-terminal ORD. Nevertheless, ORP2, as well as short variants of ORP1, ORP4 and ORP9, are devoid of the PH domain and, in some cases, the FFAT motif too. Members of subfamily IV (ORP5 and ORP8) and subfamily VI (ORP10 and ORP11) have no FFAT motif. ORP5 and ORP8 contain a transmembrane segment. ORP1L has an N-terminal AR domain. Importantly, a conserved EQVSHHPP sequence was identified in the ORD, which became the signature of ORP/Osh family.

The idea that OSBP controls sterol metabolism was progressively ruled out. Instead, it was proposed that OSBP regulates cellular sterol distribution. Sterol is scarce in the ER (<5 mol% of lipids) but represents up to 40 mol% of lipids in the *trans-*Golgi and the PM (Mesmin and Maxfield, 2009). The maintenance of this sterol concentration gradient in eukaryotic cells was suggested to mostly rely on non-vesicular routes (DeGrella and Simoni, 1982; Urbani and Simoni, 1990; Baumann et al., 2005). This prompted a search for specialized LTPs that were able to move sterol between organelles. OSBP was a plausible candidate (Raychaudhuri and Prinz, 2010) along with Osh proteins whose absence was found to alter ergosterol metabolism and distribution in yeast (Beh et al., 2001; Beh and Rine, 2004). The fact that OSBP has a dual ability to bind the ER and *trans-*Golgi membrane reinforced this hypothesis. Indeed, OSBP can populate ER-Golgi contact sites (Ladinsky et al., 1999; Marsh et al., 2004) which are thought, like other contact sites, to be hot spots for lipid transfer (Olkkonen and Levine, 2004).

**FIGURE 3:**
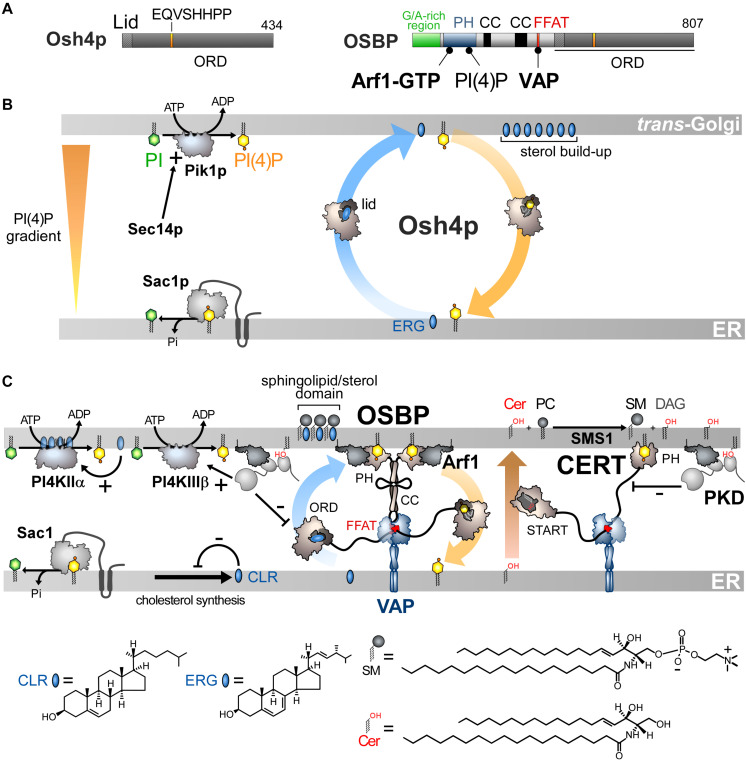
Sterol/PI(4)P exchangers supply the Golgi membrane with sterol and coordinate SM synthesis with this delivery process at ER-Golgi contact sites. **(A)** Domain organization of Osh4p and OSBP. Osh4p contains only an ORD. The position of the lid and EQVSHHPP signature in this domain is indicated. The interaction between the domains of OSBP with PI(4)P and other proteins are shown by black lines. G/A, glycine/alanine; PH, pleckstrin homology domain; CC, coiled-coil region. **(B)** In yeast, ergosterol is synthesized in the ER. Osh4p transfers ergosterol from the ER to the *trans*-Golgi/post-Golgi membrane and PI(4)P in the backward direction. Pik1p ensures the ATP-dependent phosphorylation of PI into PI(4)P and Sec14p enhances this process. At the ER, Sac1p hydrolyses PI(4)P into PI. This sustains a PI(4)P gradient that drives the transport of sterol, thereby promoting the build-up of sterol in the acceptor membrane, generating a sterol gradient at the ER/Golgi interface. **(C)** OSBP interacts via its PH domain with PI(4)P and Arf1-GTP and, through its FFAT motif, with ER-resident VAP receptors. As such, OSBP can bridge the ER and the *trans-*Golgi membrane and contribute to creating ER-Golgi contact sites. PI4KIIIβ is close to OSBP and provides PI(4)P for the sterol/PI(4)P exchange process. The delivery of sterol to the Golgi membrane can indirectly enhance the activity of a second PI 4-kinase (PI4KIIα). OSBP also facilitates the recruitment of CERT by PI(4)P and VAP receptors. CERT releases ceramide into the *trans*-Golgi, thereby promoting the synthesis of SM and DAG. DAG recruits the PKD which promotes, by phosphorylation, PI(4)P synthesis but negatively regulates the Golgi localisation of OSBP and CERT. The consumption of PI(4)P and production of DAG would trigger the disassembly of ER-Golgi contact sites and stop sterol transfer and SM production. Cer, ceramide; SM, sphingomyelin; ERG, ergosterol; CLR, cholesterol. The chemical structures of sterol, SM and ceramide are indicated. The structure of the other lipids is described in Figure 2.

Osh4p is one of the simplest ORPs/Osh proteins, as it only consists of an ORD (Figure 3A). Im and co-workers solved its crystal structure and revealed that it corresponds to an incomplete β-barrel with a deep pocket to host one sterol molecule (Im et al., 2005). This pocket is closed by an N-terminal lid of ∼ 30 amino-acids. The sterol is in a head-down orientation with its 3-hydroxyl group interacting with polar residues at the bottom of the pocket. The rest of the sterol is in contact with the pocket wall and inner side of the lid, stabilizing the closed conformation of Osh4p. The EQVSHHPP signature is recognizable in the structure: the two histidine residues followed by two proline residues are positioned in a β-hairpin that overhangs the entrance of the sterol-binding pocket. Data suggested that the lid opens when Osh4p delivers sterol to the membrane and closes when Osh4p extracts sterol. Osh4p and OSBP were found to transfer sterol between artificial membranes (Raychaudhuri et al., 2006; Ngo and Ridgway, 2009), supporting the notion that ORP/Osh proteins are sterol transporters (Levine, 2005). However, this idea was subsequently challenged. *In vitro*, Osh4p is a slow sterol transporter (Raychaudhuri et al., 2006) and several Osh proteins display no sterol-transfer activity (Schulz et al., 2009). It is also not certain that Osh proteins transfer sterol in yeast, notably at the ER/PM interface (Schulz et al., 2009; Georgiev et al., 2011). Finally, there are puzzling links between Osh proteins and PI(4)P. Indeed, silencing Osh4p bypasses the requirement for Sec14p meaning that yeasts devoid of Sec14p survive if Osh4p is lacking (Fang et al., 1996). In fact, Osh4p counteracts Sec14p by downregulating the Golgi PI(4)P pool (Fairn et al., 2007). Moreover, Osh4p regulates exocytosis (Fairn et al., 2007; Alfaro et al., 2011), which relies on the PI(4)P-dependent genesis of post-Golgi trafficking vesicles. In addition, Osh3p was found to downregulate PI(4)P at ER/PM interface (Stefan et al., 2011). This suggested that the Osh proteins, in addition to, or instead of transferring sterol, had functions related to PIPs. Our team eventually found that Osh4p sequesters PI(4)P and sterol in a mutually exclusive manner. We solved the structure of the 1:1 Osh4p-PI(4)P complex and determined that the sterol-binding pocket hosts the PI(4)P acyl chains, whereas the cationic residues that define an adjacent and shallow pocket under the lid, recognize the PI(4)P headgroup. These residues belong to the helix α7 and the EQVSHHPP signature. The lid covers the glycerol moiety of PI(4)P.

These findings qualified Osh4p as a sterol/PI(4)P exchanger (de Saint-Jean et al., 2011) and led us to propose a model based on the observation that PI(4)P gradients exist in the cell (Figure 3B). PI(4)P is prominent in the Golgi and the PM (Di Paolo and De Camilli, 2006; Strahl and Thorner, 2007) but absent from the ER due to Sac1 (Foti et al., 2001; Faulhammer et al., 2007). Consequently, a steep PI(4)P concentration gradient exists both at the ER/Golgi and ER/PM interface. Because genetic interactions exist between OSH4, SEC14, SAC1 and PIK1 genes and given that Osh4p locates to the *trans*-Golgi/post-Golgi level (Li et al., 2002; Fairn et al., 2007), we proposed that Osh4p exploits a PI(4)P gradient at the ER/Golgi interface to vectorially export sterol from the ER to the Golgi, by sterol/PI(4)P exchange cycles. Within a cycle, Osh4p extracts a sterol molecule from the ER membrane, exchanges sterol for PI(4)P at the PM, and then delivers PI(4)P to the ER membrane where PI(4)P is converted into PI. When sterol is released by Osh4p in the *trans*-Golgi membrane, it is not re-extracted by the protein because PI(4)P competes with it. Conversely, once PI(4)P is delivered in the ER membrane, it encounters Sac1p and is hydrolyzed, meaning that Osh4p has no other option but to capture sterol. Thus, the presence of a PI(4)P gradient maintained by a distant PI 4-kinase and PI(4)P phosphatase can efficiently drive non-stop exchange cycles and the accumulation of sterol in the *trans*-Golgi membrane.

This model explains why Osh4p downregulates the cellular PI(4)P level (Fairn et al., 2007) and how Sac1p, although it resides at the ER, can get access to its substrate. *In vitro* measurements supported this model, showing that Osh4p is 10-times more efficient as an exchanger than as a mere transporter (Moser Von Filseck et al., 2015b). Any mutation that compromises the recognition and transfer of one ligand by Osh4p impacts the transfer of the other ligand. Thus, the Osh4p structure encodes a tight coupling between forward sterol and backward PI(4)P transfer between membranes. Moreover, Osh4p can create and maintain a sterol gradient between two membranes by dissipating a pre-existing PI(4)P gradient. These data and the fact that the residues that recognize PI(4)P are strictly conserved in ORPs/Osh proteins suggested that all of these were lipid exchangers driven by PI(4)P (Moser von Filseck and Drin, 2016).

It is still unclear how the cellular function of Osh4p relates to its exchange activity. Osh4p is involved in polarized exocytosis by regulating the genesis and fate of vesicles trafficking from the Golgi to the PM. Likely, Osh4p provisions the TGN with sterol that coalesces with sphingolipids to promote the budding of exocytotic vesicles. By consuming PI(4)P to deliver sterol, Osh4p might also control Drs2p, a PI(4)P-dependent PS flippase, and other PI(4)P-dependent proteins critical for vesicle genesis. Osh4p removes PI(4)P from exocytic vesicles, making them competent to dock with the PM (Ling et al., 2014; Smindak et al., 2017) and possibly augments their sterol content, by sterol/PI(4)P exchange, to help the fusion process (Smindak et al., 2017). The sterol likely originates from the ER, rather than the PM as proposed recently (Smindak et al., 2017), since Osh4p extracts sterol more efficiently from fluid, ER-like than rigid, PM-like membranes (Moser Von Filseck et al., 2015b). Given its abundance (32.10^3^ copies per cell) (Ghaemmaghami et al., 2003) and its sterol transfer rate driven by PI(4)P (>10 sterols.min^–1^ per protein), Osh4p could supply the TGN and post-Golgi vesicles with 32.10^4^ sterol molecules per minute. During asymmetric division, a process that lasts 2 h, Osh4p could deliver up to 60% of 60.10^6^ sterol molecules that are required, along with other lipids, for doubling the PM surface of the mother cell (Sullivan et al., 2006). This model might explain why changes in Osh4p levels or of its affinity for ligands alter the levels or transversal repartition of sterol in the Golgi membrane and the PM (Proszynski et al., 2005; Georgiev et al., 2011), or repress exocytosis (Alfaro et al., 2011). It is unclear whether Osh4p exclusively uses the Golgi PI(4)P pool. At steady state, yeast PI(4)P levels are low (80,000 PI(4)P molecules per cell with 50% at the Golgi level) (Fairn et al., 2007). This does not indicate how much PI(4)P is hydrolyzed and regenerated over time. As previewed by Stefan and co-workers (Stefan et al., 2011) and measured in human cells (Mesmin et al., 2017), a fast PI(4)P turnover probably exists in yeast to drive sterol transfer.

## Sterol/PI(4)P Exchange at ER/Golgi Contacts Synchronizes Lipid Flows

Finding that Osh4p acts as lipid exchanger allowed us to determine that OSBP works in the cell as a LTP but also as a protein that orchestrates several lipid flows at ER-Golgi contact sites (Mesmin et al., 2013). OSBP dimerizes via coiled-coil regions and bridges the ER with *trans*-Golgi, via its FFAT motif and PH domain (Figures 3A,C). The OSBP ORD transfers then sterol from the ER to the Golgi. This is coupled with a backward transfer of PI(4)P to the ER membrane where PI(4)P is hydrolysed (Figure 3C). OSBP massively contributes to the ER-to-TGN transfer of sterol by consuming ∼50% of the whole cellular PI(4)P pool (Mesmin et al., 2017). Thus, like Osh4p, the exchange process converts the energy of a PI(4)P gradient into a forward transfer of sterol to the Golgi apparatus (Figure 3C).

Importantly, sterol/PI(4)P exchange plays a second, critical role as it controls the residence time of OSBP and other LTPs in contact sites. Indeed, PI(4)P serves as an anchor point on the Golgi membrane, along with Arf1-GTP, for the PH domain of OSBP. Once OSBP is docked on this organelle, sterol/PI(4)P exchange starts, and PI(4)P is consumed. Consequently, PI(4)P levels decrease, forcing OSBP to disengage from contact sites. One can wonder what justifies such a mechanism since the exhaustion of PI(4)P would arrest the transfer activity of the ORD even if OSBP remains attached to the Golgi surface. In fact, this negative feedback loop synchronizes sterol and sphingolipid abundance in the Golgi membrane, which is crucial for the biogenesis of non-coated vesicles (Duran et al., 2012). Early investigations showed that, intriguingly, OSBP, in addition to VAP, influences the association of the CERT protein with the Golgi complex (Perry and Ridgway, 2006). CERT carries ceramide from the ER to the *trans*-Golgi (Hanada et al., 2003), where ceramide is transformed into sphingomyelin (SM). It is noteworthy that CERT has the same molecular configuration as OSBP. It contains a START domain instead of an ORD to transfer ceramide but, like OSBP, it bridges the ER and the Golgi membrane via a FFAT-VAP interaction and a PI(4)P-specific PH domain, respectively (Hanada et al., 2003; Kawano et al., 2006). Thus PI(4)P can recruit both OSBP and CERT at ER-Golgi contact sites to simultaneously switch on sterol and ceramide transfer. Inversely, a complete consumption of PI(4)P stops these two processes (Figure 3C; Mesmin et al., 2013, 2017; Capasso et al., 2017). Interestingly, the Golgi PI 4-kinase PI4KIIIβ colocalizes with OSBP, meaning that the PI(4)P source is near the sterol/PI(4)P exchange machinery (Mesmin et al., 2017). This spatial proximity likely arises from the fact that PI4KIIIβ is recruited onto the membrane by Arf1 (Godi et al., 1999). Secondly, PI4KIIIβ docks onto membrane domain whose features (e.g., low lipid-packing) are suited for the recruitment of OSBP by PI(4)P and Arf1. A secondary, more distant PI(4)P source is positively tuned by sterol delivery (Mesmin et al., 2017). Indeed, the presence of sterol activates a palmitoyltransferase that grafts lipid tails to PI4KIIα and thereby ensures its docking to the Golgi (Lu et al., 2012). This second source of PI(4)P elicits the recruitment of CERT to the ER-Golgi contact sites (Banerji et al., 2010) and OSBP in sterol-rich areas (Mesmin et al., 2017). The synthesis of SM, known to promote the thermodynamic trapping of sterol, might assist the PI(4)P-driven delivery of sterol in the Golgi membrane, as measured *in vitro* with Osh4p (Moser Von Filseck et al., 2015b). Possibly, Nir2 promotes the activity of OSBP and CERT by assisting the synthesis of Golgi PI(4)P (Peretti et al., 2008).

Additional layers of regulation exist and have been recently reviewed (Mesmin et al., 2019). In brief, the conversion of ceramide into SM gives DAG as a by-product. DAG acts as a signaling lipid that cooperates with Arf1-GTP to recruit Protein Kinase D (PKD) (Pusapati et al., 2009), which can then get access to CERT and, through phosphorylation, limits its association with PI(4)P (Prashek et al., 2017; Sugiki et al., 2018). PKD also enhances PI4KIIIβ activity (Hausser et al., 2005) but more permanently that of OSBP. Overall, this results in a net depletion of Golgi PI(4)P. Consequently, OSBP and CERT disengage from contact sites and cease to function (Capasso et al., 2017). All these mechanisms, connected to the sterol/PI(4)P cycle acting as a central timing belt, synchronize sterol and ceramide fluxes, ensuring co-enrichment of both lipids at the Golgi, while regulating DAG and PI(4)P levels. This cellular logistic hub precisely controls the lipid composition of the Golgi membrane, and thereby its secretory function (Duran et al., 2012).

## OSBP Plays Key Roles in Other Subcellular Regions and is Involved in Pathologies

Functional links exist between OSBP and the endosomal/lysosomal compartment. First, OSBP populates ER-endosomes contact sites to transfer endosomal PI(4)P for clearance by Sac1, thereby controlling WASH-dependent actin nucleation on endosomes and the function of the retromer, a protein coat responsible for endosome-to-Golgi traffic (Dong et al., 2016). Secondly, OSBP is engaged in ER-lysosome contacts to convey sterol to the limiting membrane of lysosomes. This enables mTORC1, a master regulator of cell growth, to be activated on lysosome surface. Thus, OSBP “informs” mTORC1 on cellular sterol availability to launch downstream programs (Lim et al., 2019). Whether these two functions rely on sterol/PI(4)P exchange cycles is unclear. OSBP also connects recycling endosomes to the TGN by interacting with the endosomal RELCH-Rab11 complex to transfer sterol from the first to the second organelle (Sobajima et al., 2018). We do not know whether a PI(4)P level gradient exists at the endosome/TGN interface to fuel this process.

Human pathogens such as enteroviruses (e.g., rhinovirus or poliovirus) or hepatitis C virus (HCV) impose large changes on the morphology and lipid content of host cell membranes as they replicate. All these viruses trigger an overproduction of PI(4)P to remodel compartments into replication organelles that host supracomplexes, combining viral and host proteins, able to replicate the viral genetic material (Romero-Brey and Bartenschlager, 2014). PI4KIIIβ is hijacked by the 3A protein of enteroviruses for overproducing PI(4)P at the Golgi (Hsu et al., 2010) whereas HCV takes control of PI4KIIIα to boost PI(4)P synthesis at the ER (Berger et al., 2009). This PI(4)P overproduction diverts the sterol/PI(4)P exchange activity of OSBP which delivers sterol in high quantities to the replication organelles (Roulin et al., 2014; Wang et al., 2014; Strating et al., 2015). Presumably, sterol, together with sphingolipids, creates an ideal membrane environment for the replication machinery. Of note, Nir2 also contributes to the replication of hepatitis C virus (HCV) by promoting the enrichment of viral replication organelle with PI(4)P (Wang and Tai, 2019). Likewise, PITPβ is a host factor required for the replication of human viruses (Roulin et al., 2014; Ishikawa-Sasaki et al., 2018).

Some drugs, belonging to a class of molecules called ORPhilins, exert an antiviral activity by targeting OSBP. OSW1 binds with a nanomolar affinity to the ORD of OSBP and blocks its exchange activity (Mesmin et al., 2017), thereby inhibiting the replication of enteroviruses (Albulescu et al., 2015) or HCV (Wang et al., 2014). Itraconazole also stops viral replication by binding to the OSBP ORD (Strating et al., 2015). Interestingly, many ORPhilins were initially identified as compounds that inhibited the growth of human cancer cell lines by targeting OSBP (Burgett et al., 2011).

## PS/PI(4)P Exchangers Represent a New Class of Heterotypic Lipid Exchangers in ORP/Osh Family

**FIGURE 4:**
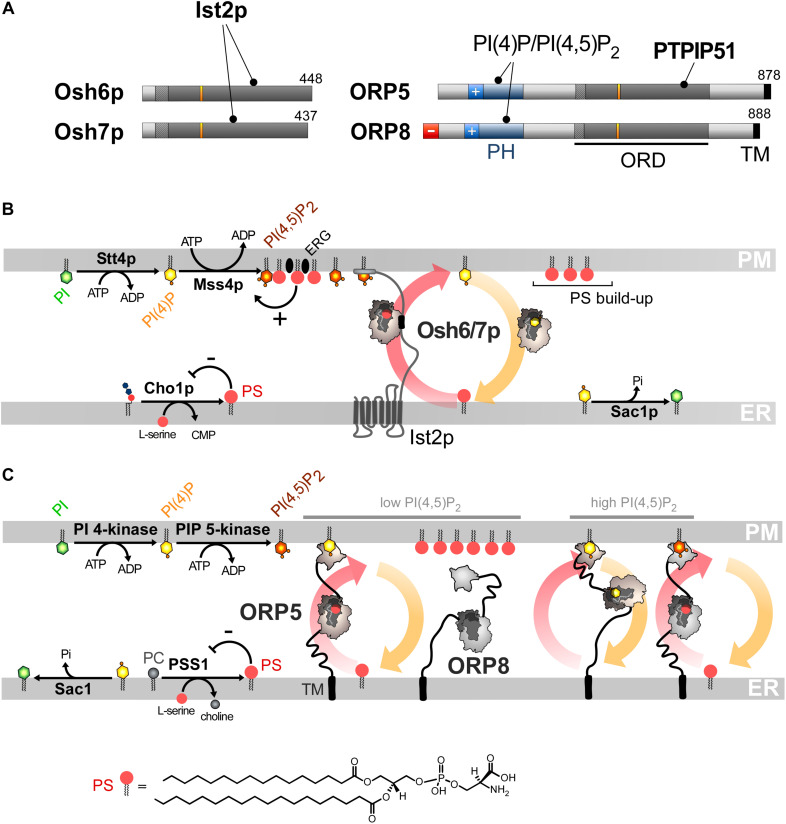
PS/PI(4)P exchange at the ER/PM interface. **(A)** Domain organization of Osh6/7p and ORP5/8. The interaction between Osh6/7p or ORP5/8 with PIPs and other proteins are shown by black lines. TM, transmembrane domain. **(B)** PS is made by Cho1p from CDP-DAG at the ER. It is transferred by Osh6p/7p to the PM by using a PI(4)P gradient sustained by Stt4p and Sac1p. Osh6p interacts with the cytosolic tail of Ist2p to partially occupy ER-PM contact sites and transfer PS. The synthesis of PI(4,5)P_2_ by Mss4p enables the recruitment of Ist2p to the PM. The activity of Mss4p is positively tuned when it associates with both PS- and sterol-rich domains. **(C)** ORP5/8 are anchored to the ER by a TM domain and bind to the PM PI(4)P with PH domains. They must bind the ER and a second membrane to exchange lipids. When the PI(4,5)P_2_ levels are high, ORP8 is recruited together with ORP5 to supply PS and decrease PI(4)P level to limit PI(4,5)P_2_ synthesis. At low PI(4,5)P_2_ levels, only ORP5 docks to the PM and supplies PS unless the PM PI(4)P pool is exhausted. PSS1 synthesizes PS and its activity is inhibited by the end-product of the reaction. The chemical structure of PS is represented in panel **C** whereas the structure of the other lipids is shown in Figures 2, 3.

Phosphatidylserine accounts for 2–10% of total membrane lipids (Daum et al., 1999; Leidl et al., 2008; Ejsing et al., 2009; Sampaio et al., 2011). It is distributed along a gradient between the ER and the PM, more specifically its cytosolic leaflet, where it represents 5–7% and up to 30% of glycerophospholipids, respectively (Vance and Steenbergen, 2005; Leventis and Grinstein, 2010). Both this build-up and the asymmetric distribution of PS in the PM are critical for signaling pathways. Indeed, given its negative charge, PS facilitates the recruitment and activity of signaling proteins including Akt, PKC and phospholipases (Leventis and Grinstein, 2010; Huang et al., 2011). PS must be actively transported to the PM since, like sterol, it originates from the ER (Vance and Tasseva, 2013). Little was known about how this is achieved (Leventis and Grinstein, 2010) until the finding that, in yeast, Osh6p and its close homolog Osh7p (Figure 4A), selectively sequester and convey PS from the ER to the PM (Maeda et al., 2013). Structural analyses explained why Osh6p is attuned to specifically trap PS. Subsequently, we established that PI(4)P is the second ligand of Osh6/7p and that Osh6p efficiently exchanges PS for PI(4)P between membranes. In yeast, Osh6p does not transfer PS if its ability to recognize PI(4)P is disabled. Likewise, silencing Sac1p blocks Osh6p activity as the PI(4)P gradient at the ER/PM interface no longer exists. We concluded that Osh6/7p execute PS/PI(4)P exchange to directionally transfer newly synthesized PS to the PM (Figure 4B; Moser Von Filseck et al., 2015a).

Osh6/7p are cytosolic but also locate to ER-PM contact sites (Schulz et al., 2009; Maeda et al., 2013). Recently, we showed that this is due to their interaction with the ER-residing Ist2p protein (D’Ambrosio et al., 2020). Ist2p is a homolog of Ca^2+^-activated lipid scramblases (Wolf et al., 2012, 2014; Brunner et al., 2014) and contributes to scaffolding yeast ER-PM contacts (Manford et al., 2012; Collado et al., 2019; Hoffmann et al., 2019). Its tethering capacity relies on a long and disordered cytosolic tail whose highly cationic C-terminal end binds to the PI(4,5)P_2_ present in the PM. Osh6p recognizes a short motif within this linker and this is critical for its exchange activity (Figure 4B), in terms of speed and maybe accuracy. It is unclear whether Ist2p has a scramblase activity (Lee et al., 2018) and whether this is critical for the activity of Osh6/7p.

ORP5 and ORP8 contain an ORD, a PH domain to interact with the PM and are anchored to the ER via a C-terminal transmembrane segment and not by interacting with VAP (Yan et al., 2008; Du et al., 2011; Figures 4A,C). The ORD of ORP5 and ORP8 is the most closely related to that of Osh6p and Osh7p and the ORP5 ORD was found to capture PS (Maeda et al., 2013). A following study showed that ORP5 and ORP8 colonize ER-PM contact sites to deliver PS in the PM by PS/PI(4)P exchange, driven by the synthesis of PI(4)P at the PM and its hydrolysis at the ER (Chung et al., 2015). Thus, PS/PI(4)P exchange is an evolutionarily conserved mechanism.

## Interplay Between PS/PI(4)P Exchange and PIP Metabolism at the ER/PM Interface

Interestingly, ORP5/8 downregulate PM PI(4,5)P_2_ levels and, challenging previous structural analyses of ORD (de Saint-Jean et al., 2011; Manik et al., 2017), it has been reported that ORP5/8 could use PI(4,5)P_2_ as a counterligand, instead of PI(4)P, to provide the PM with PS (Ghai et al., 2017). Yet, another report suggested that PI(4)P is the real ligand (Sohn et al., 2018) and that the PI(4,5)P_2_ level solely decreases as PI(4)P is the substrate of the PI(4)P 5-kinase (PIP5K) enzyme. In other words, PI(4)P transfer counteracts PI(4,5)P_2_ synthesis.

As observed for OSBP (Mesmin et al., 2013) an interdependence exists between the mobilization of ORP5 and ORP8 in contact sites and their exchange activity, yet in a more sophisticated manner. The PH domain of ORP5/8 recognizes PI(4)P and PI(4,5)P_2_ (Ghai et al., 2017; Lee and Fairn, 2018; Sohn et al., 2018). A short cationic region, appended to the PH domain, helps ORP5 and ORP8 to associate with the PM (Lee and Fairn, 2018; Sohn et al., 2018). However, these proteins are not alike: ORP8 weakly associates with the PM, in a more PI(4,5)P_2_-dependent fashion, compared to ORP5. This is determined by the features of the PH domain and the presence of an anionic N-terminal end that limits its association with the anionic surface of the PM. These differences between the sensory aptitudes of the PH domain of ORP5 and ORP8 form the basis of a rheostat mechanism that exquisitely regulates PI(4)P, PI(4,5)P_2_ and PS levels at the PM. A decrease in PI(4)P or PI(4,5)P_2_ levels would mainly reduce ORP5 transfer activity to restore, in turn, optimal PIP levels. In contrast, a rise in PI(4,5)P_2_ levels would mobilize ORP8 at the PM, in addition to ORP5, to transfer more PI(4)P to the ER, thereby preventing extra PI(4,5)P_2_ production (Figure 4C).

Remarkably, the presence of PI(4)P alone in the PM is insufficient for the yeast PI(4)P 5-kinase Mss4p to make PI(4,5)P_2_. Osh6/7p, through PS transfer and the help of other Osh proteins (likely Osh4p), are required to create domains in the PM, made of unsaturated PS and ergosterol, to which Mss4p efficiently binds to exert its activity (Nishimura et al., 2019). Thus, PI(4)P-driven exchange cycles regulate PI(4,5)P_2_ production negatively and positively, by reducing the PI(4)P availability and by delivering PS and sterol, respectively. Together, these processes precisely tune the lipid composition and PIPs-based signaling competence of the PM.

Collectively, these data revealed that ORP/Osh proteins ensure the accumulation of PS in the PM, while firmly controlling PI(4,5)P_2_ levels in that membrane. Furthermore, PS/PI(4)P exchange tightly connect ER and PM lipid metabolism. Pharmacological inhibition of PI4KIIIα, which provides PI(4)P to ORP5/8 at the PM, lowers PS synthesis at the ER (Sohn et al., 2016). The reason is that PSS1 and PSS2 enzymes, which make PS by swapping the head of PC and PE, respectively, for serine (Vance and Tasseva, 2013) are inhibited by the end-product of the reaction (Kuge et al., 1998). Thus, with no PI(4)P gradient to drive PS export out of the ER, PS inhibits its own production. Remarkably, in yeast, silencing the equivalent kinase, Stt4p reduces the cellular PS level by limiting the activity of Pss1p (*a.k.a.* Cho1p) that generates PS from CDP-DAG and serine at the ER (Tani and Kuge, 2014). Moreover, silencing Sac1p or limiting Osh6/7p activity has the same effect (D’Ambrosio et al., 2020). Thus, if PS/PI(4)P exchange are stopped, PS synthesis is repressed due to elevated PS levels at the ER. Of note, missense mutations of PSS1, which render the enzyme insensitive to feedback inhibition by PS and lead to PS overproduction, cause Lenz-Majewski syndrome which is characterized by a generalized craniotubular hyperostosis and intellectual disability (Sousa et al., 2014). Exactly how the development of this syndrome relies on the alteration of PS metabolism is unclear. In cells in which PS is overproduced, ORP8 is absent from ER-PM contacts and weak PI4P clearance from the PM is detected (Sohn et al., 2016). These anomalies, of which the mechanistic bases are unclear, suggest that defects in PIP metabolism might also be involved in the Lenz-Majewski syndrome.

## Roles of PS/PI(4)P Exchange in Other Cellular Regions and in Diseases

Phosphatidylserine is also exported from the ER to the mitochondrion and reaches the inner membrane of this organelle for decarboxylation into PE (Vance and Tasseva, 2013). PS transfer occurs in zones of close apposition between the ER and the outer mitochondrial membrane. In yeast, this transfer would be mediated by the ERMES complex. Ups2-Mdm35p (SLMO2-TRIAP1 in humans) then transfers PS to the inner membrane (Tamura et al., 2019). Interestingly, ORP5/8 reside at ER-mitochondria contact sites and help to preserve the morphology and respiratory function of mitochondria, possibly by PS import (Galmes et al., 2016). ORP5 interacts with PTPIP51 (protein tyrosine phosphatase interacting protein-51). This outer mitochondrial membrane protein associates with VAP-B and helps to anchor mitochondria to the ER to support IP3 receptor-mediated delivery of Ca^2+^ from ER stores to mitochondria and its metabolism (Stoica et al., 2014; Gomez-Suaga et al., 2019). It is unclear whether ORP5 operates PS/PI(4)P exchange since it is not known whether the mitochondrial outer membrane contains PI(4)P. However, this membrane is surprisingly rich in PI, suggesting that PIPs can be synthesized there (Pemberton et al., 2020; Zewe et al., 2020). ORP5 also localizes to ER-LD contact sites. ORP5 associates with the LD monolayer via its ORD and transfer PS in exchange for PI(4)P made by PI4KIIα. ORP5 seems mandatory for the growth of LDs but it is unclear why since the role of PS itself is elusive. Possibly, given its low levels in the monolayer, PS has no structural role but rather a signaling/regulatory function (Du et al., 2020).

Given that ORP5/8 moves lipids around in diverse subcellular regions, one can anticipate that any alteration of their activity results in cell dysfunctions. Supporting this idea, reports have shown that ORP5 expression is linked to increased cancer cell invasion and metastasis. For instance, the invasiveness of pancreatic cancer cells is enhanced by ORP5 overexpression and reduced by ORP5 depletion (Koga et al., 2008). Moreover, analysis of clinical samples suggested that poor prognosis in human pancreatic cancer relates to high expression levels of ORP5 (Koga et al., 2008). ORP5 is also highly expressed in lung tumor tissues, notably those of metastasis-positive cases (Nagano et al., 2015). Furthermore, ORP5 promotes the proliferation and migration of HeLa cells, and this depends on its ability to transfer lipids. Of note, ORP5 positively regulates the mTORC1 complex, which plays a key role in activating cell proliferation and survival (Du et al., 2018). A possible reason is that the activation of Akt, a major upstream effector of mTORC1, strongly depends on its recruitment to the PM by PS (Huang et al., 2011). This supports the idea that any deregulation of ORP5 can distort the signaling capacity of the cell by changing the lipid content of the PM. Corroborating this, a lack of ORP5/8 activity was found to result in lower PS abundance in the PM, reducing the oncogenicity of K-Ras, a signaling protein that is frequently mutated in human cancers (Kattan et al., 2019).

## ORP2 Executes a New Type of Exchange

**FIGURE 5:**
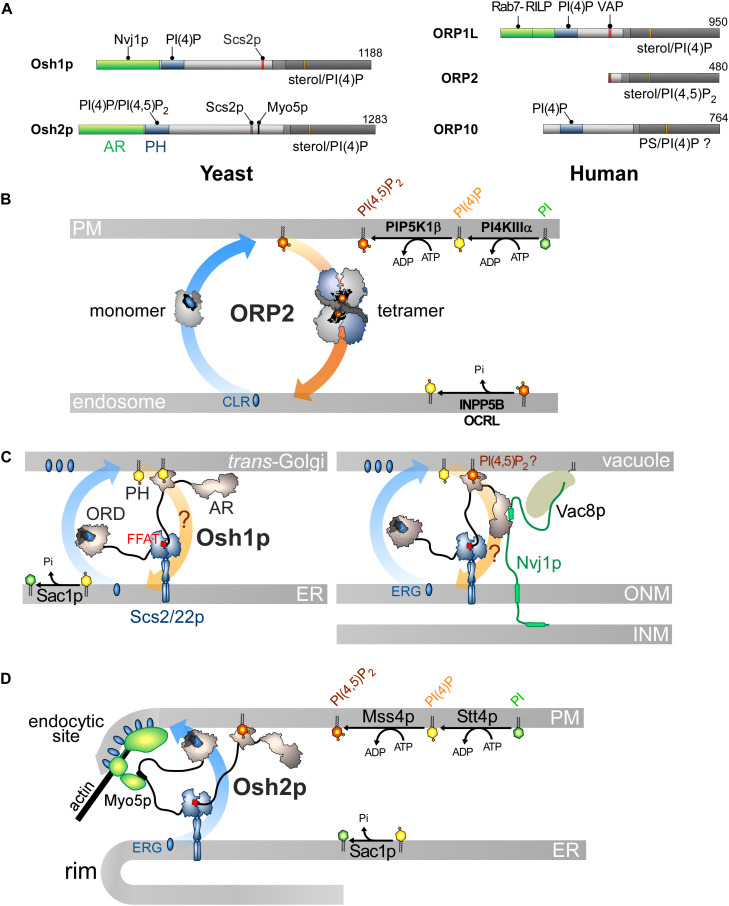
Other ORP/Osh proteins act or likely act as lipid exchangers. **(A)** Domain organization of yeast Osh1p and Osh2p, and human ORP1L, ORP2 and ORP10. The interaction between Osh proteins and ORPs with PIPs and other proteins are shown by black lines. AR, ankyrin repeat domain. **(B)** Model for ORP2-mediated cholesterol/PI(4,5)P_2_ exchange. ORP2 transfers cholesterol forward from the endosomal compartment to the PM as a monomer and transfers PI(4,5)P_2_ backward as a tetramer. **(C)** Osh1p might occupy ER-Golgi contacts by simultaneously bridging the ER and the Golgi membrane via its PI(4)P-binding PH domain and by interacting with Scs2/22p via its FFAT motif; it would exchange sterol for PI(4)P with its ORD. By alternatively binding to Nvj1p via its AR domain, Osh1p occupies NVJs where it might also function as an exchanger. Whether its PH domain contributes to recruiting Osh1p to vacuolar membrane is unclear. **(D)** Osh2p locates to ER-PM contact sites and binds to Myo5p for delivering ergosterol at endocytic sites, maybe by sterol/PI(4)P exchange. ERG, ergosterol; CLR, cholesterol.

ORP2 is expressed ubiquitously in mammalian tissues and consists of an ORD preceded by an FFAT motif (Figure 5A; Laitinen et al., 2002; Loewen et al., 2003). It is able to host cholesterol, oxysterols (notably 22-HC), PI(4)P or PI(4,5)P_2_ (Wang et al., 2019). ORP2 is cytosolic but prone to locate to the Golgi apparatus, the ER, LDs and the PM (Laitinen et al., 2002; Hynynen et al., 2009; Wang et al., 2019). ORP2 is likely to intervene in TAG metabolism at ER-LD contact sites (Weber-Boyvat et al., 2015b). This would rely on its interaction with VAP, but it is unclear how it targets the LD surface. Other studies showed that overexpressing ORP2 reduces sterol esterification at the ER and increases sterol efflux out the cell, suggesting that ORP2 exports sterol to the PM (Hynynen et al., 2005). It has recently been shown that ORP2 supplies the PM with sterol in exchange for PI(4,5)P_2_ in liver cells (Wang et al., 2019). Moreover, a lack of ORP2 provokes an enrichment of LEs with sterol at the expense of the PM. This suggests that ORP2 picks up sterol from endosomes and exploits a PI(4,5)P_2_ gradient at endosome/PM interface to deliver sterol in the PM. This gradient would be sustained by the synthesis of PM PI(4,5)P_2_ and its degradation into monophosphorylated PI at the endosome surface by 5-phosphatases INPP5B and OCRL. In terms of the energy budget, one ATP is consumed to move one sterol molecule, as proposed for sterol/PI(4)P exchangers (Figure 5B).

However, the sterol/PI(4,5)P_2_ cycle would be different: ORP2 would transport sterol as a monomer but transfer PI(4,5)P_2_ as a tetramer, but the advantage of this state is unclear. Moreover, the fact that ORP2 transfers PI(4,5)P_2_ is surprising. Indeed, previous structural analyses suggested that an ORD is unable to properly accommodate PI(3)P, PI(5)P or PI(4,5)P_2_ due to steric hindrance that would be caused by a phosphate group at position 3 or 5 of the inositol ring (de Saint-Jean et al., 2011; Manik et al., 2017). Yet it is possible to solve the crystal structure of ORP2 in 1:1 complex with PI(4,5)P_2_. Its inositol ring nests into the pocket with a ∼180° rotation compared with that of the PI(4)P headgroup in other ORD structures (de Saint-Jean et al., 2011; Tong et al., 2013; Moser Von Filseck et al., 2015a; Manik et al., 2017). Two conformations of ORP2 were resolved and one of the two corresponds to the conformation adopted by the protein in its tetrameric state. Intriguingly, the lid is systematically open and one of the acyl chains of PI(4,5)P_2_ is outside the pocket. Thus these structures differ significantly from the ORD structures in which the ligand — PI(4)P, sterol or PS — is perfectly inserted and shielded by the lid (Im et al., 2005; de Saint-Jean et al., 2011; Maeda et al., 2013; Moser Von Filseck et al., 2015a; Manik et al., 2017). *In vitro* assays did not clearly show that ORP2 reciprocally exchanges one sterol for one PI(4,5)P_2_ molecule as established for the sterol/PI(4)P activity of Osh4p (Moser Von Filseck et al., 2015b; Wang et al., 2019). One can wonder whether these ORP2-PI(4,5)P_2_ complexes are predominant in the cell and also assume that ORP2 functions as a sterol/PI(4)P exchanger. Indeed, ORP2 extracts PI(4)P from membranes *in vitro* and downregulates both PM PI(4)P and PI(4,5)P_2_ levels (Wang et al., 2019). As observed for ORP5/8 (Sohn et al., 2018), the regulation of PM PI(4,5)P_2_ levels by ORP2 might be primarily caused by its ability to execute sterol/PI(4)P exchange and thereby control PI(4)P levels. Moreover, a difference in PI(4)P concentration at the endosome/PM interface might be sufficient to drive sterol transfer. ORP2 fails to exchange sterol for PI(4)P *in vitro* but these results must be analyzed carefully. The precise nature of sterol (Liu and Ridgway, 2014) or the acyl chains of a lipid ligand, for example PS (Moser Von Filseck et al., 2015a), can strongly influence the activity of ORP/Osh proteins. It is noteworthy that OSBP weakly exchanges sterol and PI(4)P *in vitro* except in a more sophisticated assay where PI(4)P is hydrolyzed by Sac1 (Mesmin et al., 2013). OSBP is possibly stalled in a PI(4)P-bound form except when PI(4)P is degraded to allow the protein to extract a new sterol molecule. Overall, it is necessary to reassess whether ORP2 functions as a sterol/PI(4)P exchanger. Likewise, it must be explored why the FFAT motif is functionally disconnected from its transfer capacity. More broadly, it must be defined whether the ability of ORP2 to transfer lipids relates to its role in ER-LD contacts and TAG metabolism, the PI 3-kinase/Akt signaling pathway (Kentala et al., 2018), ER-mitochondria crosstalk and adrenocortical steroidogenesis (Li et al., 2013; Escajadillo et al., 2016).

## Are Other ORP/Osh Proteins Lipid Exchangers?

Sequence analyses suggest that all ORP/Osh proteins can trap PI(4)P. One might thus assume that they are all exchangers that harness PI(4)P metabolism to ferry lipids in the cell, yet this is uncertain. Indeed, Osh3p, ORP6, ORP7, and ORP11 capture or likely recognize PI(4)P but are not able to trap sterol or PS and it is unclear whether they recognize a second lipid to act as exchangers. However, some ORP/Osh proteins have a dual ability to recognize sterol and PI(4)P or PS and PI(4)P and observations suggest that they exchange these lipids in the cell. A recent report suggests that ORP3 acts as a PC/PI(4)P exchanger (D’Souza et al., 2020). Other proteins are associated with a more complex picture. We discuss this in more detail below.

## Osh1p and Osh2p Likely Act as Sterol/PI(4)P Exchangers to Support Distinct Cellular Functions

Osh1p and Osh2p likely act as sterol/PI(4)P exchangers and translate this aptitude to diverse cellular outputs, owing to interactions with given partners in specific subcellular sites. Their architecture resembles that of many ORP/Osh proteins with a PH domain, an FFAT motif and a C-terminal ORD (Figure 5A; Jiang et al., 1994) but they contain an N-terminal AR domain upstream of the PH domain.

Osh1p associates with the ER by interacting with Scs2p, the yeast VAP (Loewen et al., 2003), but it has a dual cellular localization. It localizes at the Golgi apparatus, presumably at ER-Golgi contacts, owing to its PI(4)P-binding PH domain. It also occupies nuclear-vacuolar junctions (NVJs), i.e., zone of close apposition of the outer nuclear membrane (ONM) with the vacuole (Levine and Munro, 2001). This depends on the association of its AR domain with the cytosolic part of Nvj1p (Kvam and Goldfarb, 2004, 2006; Shin et al., 2020), a protein that bridges the ONM to the vacuole through an interaction with the vacuolar protein Vac8p. The PH domain likely has no major role in recruiting Osh1p to NVJs (Levine and Munro, 2001; Shin et al., 2020; Figure 5C). Structural and biochemical analyses showed that the Osh1p ORD traps sterol and PI(4)P in a competitive manner and transfers sterol between membranes (Manik et al., 2017). Whether Osh1p can execute sterol/PI(4)P exchange between membranes like Osh4p awaits examination. Osh1p regulates post-Golgi vesicular trafficking to the PM, by supplying ergosterol to the TGN (Umebayashi and Nakano, 2003). Thus, Osh1p might use the Golgi PI(4)P source to deliver sterol. At the NVJs, Osh1p maybe conveys sterol from the ONM (in continuity with the ER) to the vacuole and regulate two sterol-dependant features of this organelle, its size and dynamics (Kato and Wickner, 2001; Hongay et al., 2002; Fratti et al., 2004; Li and Kane, 2009). It is unclear whether Osh1p moves sterol by exchange for PI(4)P because it is unknown whether the vacuolar membrane harbors PI(4)P. Interestingly, the activity of Osh1p is tuned by external signals that modify its repartition between the Golgi apparatus and NVJs (Kvam and Goldfarb, 2006; Shin et al., 2020). During the log phase, Osh1p is evenly distributed between these regions, but associates only with NVJs when the cell enters a stationary phase (Levine and Munro, 2001), due to higher expression of Nvj1p (Roberts et al., 2003). Tryptophan uptake is downregulated during nutrient depletion, probably because Osh1p is not located at the Golgi complex and cannot positively tune the export of the tryptophan permease Tat2p to the PM (Kvam and Goldfarb, 2006). Osh1p also dissociates from the Golgi membrane when the cytosolic pH becomes low in response to glucose deprivation. This relies on the fact that its PH domain has less affinity for the PI(4)P headgroup which undergoes protonation in that context (Shin et al., 2020). Thus, external factors change the yeast activities by regulating where sterol is delivered by Osh1p.

Osh2p localizes at ER-PM contacts owing to its PH domain and its FFAT motif (Loewen et al., 2003; Roy and Levine, 2004; Schulz et al., 2009; Schuiki et al., 2010; Maeda et al., 2013). Ultrastructural investigations revealed that Osh2p is at the rim of cortical ER and is physically linked to endocytic invaginations (Figure 5D; Encinar del Dedo et al., 2017). Osh2p associates with Myo5p, a type-I myosin that is required for actin assembly and scission of endocytic vesicles from the PM. Corroborating *in vitro* data (Schulz et al., 2009), Osh2p was found to create sterol-rich domains at endocytic sites to assist actin polymerization. Osh2p also lowers cellular PI(4)P levels (Stefan et al., 2011), which might reflect its sterol/PI(4)P exchange activity. It is unknown whether Osh2p regulates PI(4)P and PI(4,5)P_2_ pools to coordinate sterol delivery with the assembly/disassembly of the clathrin coat and actin polymerization.

## A Complex Picture for Several ORPs

In humans, ORP1 and ORP2 define the ORP subfamily I. ORP1 exists as a long version, (ORP1L), which contains an N-terminal AR domain followed by a PH domain, an FFAT motif and a C-terminal ORD, and a shorter one, ORP1S, only consisting of the ORD (Figure 5A; Lehto et al., 2001; Loewen et al., 2003). So far, it is unclear whether these proteins act as sterol/PI(4)P exchangers, as pure sterol transporters or sterol sensors. ORP1 ORD can trap either cholesterol or PI(4)P (Vihervaara et al., 2011; Zhao and Ridgway, 2017; Dong et al., 2019) and displays some affinity for oxysterols (Suchanek et al., 2007; Vihervaara et al., 2011). ORP1L is unique amid ORPs as it docks onto late endosomes (LEs) and lysosomes (Johansson et al., 2003), by interacting, via its AR domain, with Rab7, a protein that specifically locates to these compartments (Johansson et al., 2005) and plays regulatory roles. Rab7 recruits, once in a GTP-bound state, the Rab7-interacting lysosomal protein (RILP) that in turn recruits p150^*Glued*^, a component of dynactin/dynein motor complex. Together, these proteins control the movement of LEs/lysosomes along microtubules. When cholesterol abounds in the limiting membrane of LEs/lysosomes, ORP1L encapsulates sterol and adopts a conformation that breaks its FFAT-mediated interaction with VAP (Rocha et al., 2009; Vihervaara et al., 2011). As a result, LEs/lysosomes cluster in a perinuclear area by moving toward the minus-end of microtubules. When the cellular cholesterol level decreases, ORP1L undergoes a conformational change allowing its binding to VAP and the release of the dynactin/dynein complex. Therefore, LEs/lysosomes are scattered at the cell periphery and engaged in contacts with the ER (Rocha et al., 2009; Vihervaara et al., 2011). ORP1L might also act as an LTP according to diverse and even opposite modalities. ORP1L would transfer cholesterol from the ER to the limiting membrane of LEs, when sterol is scarce (Eden et al., 2016), maybe by using an endosomal PI(4)P pool (Hammond et al., 2014), to participate in endosome maturation. Conflicting studies suggest that ORP1L conveys LDL-derived cholesterol, expelled out from LEs/lysosomes by NPC1, to the ER along its concentration gradient (Zhao and Ridgway, 2017). This transfer cannot be ensured by sterol/PI(4)P exchange, which would drive forward sterol transfer to the endosome, but depends on PI(4)P. Recent investigations dismissed the idea that ORP1L transports PI(4)P, showing instead that its sterol transport activity is enhanced by a endosomal/lysosomal PI(3,4)P_2_ pool (Dong et al., 2019). ORP1S has a cytoplasmic/nuclear distribution. It moves cholesterol from the PM to the ER and LDs (Jansen et al., 2011), but also from LEs/lysosomes to the PM, counteracting ORP1L action (Zhao et al., 2020). It is unclear how the capacity of ORP1S to shuttle sterol relates to the recognition of PI(4)P or other PIPs (Zhao et al., 2020). Overall, it remains difficult to define how ORP1S and ORP1L precisely function.

ORP3 is the best characterized member of subfamily III. It contains a PI(4)P/PI(4,5)P_2_-specific PH domain to associate with the PM (Gulyás et al., 2020), a canonical and non-canonical FFAT motif (Loewen et al., 2003; Weber-Boyvat et al., 2015a) and a C-terminal ORD that was recently shown to capture PI(4)P or PC, but neither PS nor sterol (D’Souza et al., 2020; Gulyás et al., 2020). ORP3 relocates to ER-PM contact sites upon phosphorylation by PKC following PMA treatment or agonist stimulation (Lehto et al., 2008; Weber-Boyvat et al., 2015a; Gulyás et al., 2020), a process that is synergized by an elevation of intracellular Ca^2+^ through store-operated calcium entry (SOCE) by the STIM/Orai1 complex. ORP3 might undergo a conformational change that exposes its PH domain and FFAT motif and ensures its translocation to contact sites. ORP3 activation by PKC and Ca^2+^ entry is linked to a mechanism implicated in focal adhesion dynamics. Once recruited to ER-PM contact sites, ORP3 interacts with IQSec1, a guanine nucleotide exchange factor of Arf5 to trigger focal adhesion disassembly (D’Souza et al., 2020). It is proposed that this process, associating STIM/Orai1, ORP3 and IQSec1/Arf5 occurs at the rear-front of cells to guarantee their migration (Machaca, 2020). It is unclear how this relates to reports showing that ORP3 recruits R-Ras, a small G protein that is also involved in cell adhesion and migration (Lehto et al., 2008; Weber-Boyvat et al., 2015a). Likewise, it is unknown whether the cellular function of ORP3 relies on its ability to downregulate PM PI(4)P levels and, maybe, to mediate PC/PI(4)P exchange (D’Souza et al., 2020; Gulyás et al., 2020).

ORP4 is the closest homolog of OSBP and is able to host sterol or PI(4)P via its ORD, to interact with VAP, and target PI(4)P via its PH domain (Wyles et al., 2007; Goto et al., 2012; Charman et al., 2014). However, ORP4 has distinct functions, and it is unclear whether it acts as a sterol/PI(4)P exchanger. ORP4L can partially localize to ER-Golgi contact sites, with its PH domain and through heterodimerization with OSBP to regulate Golgi PI(4)P homeostasis (Pietrangelo and Ridgway, 2018). However, ORP4 and shorter variants can interact with intermediate filaments called vimentin and remodel the vimentin network near the nucleus (Wang et al., 2002). Also, ORP4 is critical for the survival and proliferation of immortalized and transformed cells (Charman et al., 2014; Zhong et al., 2016a, b). In lymphoblastic leukemia T cells, ORP4L partly localizes on the PM where it serves as scaffolding protein for G-protein coupled receptors and PLC-β3. This triggers the production of IP3 and Ca^2+^ release from ER stores, which ensures the proliferation of macrophages and transformed T-cells (Zhong et al., 2016a, b, 2019). Possibly, ORP4L promotes PLC-β3 translocation from the nucleus to the PM (Pan et al., 2018) and extracts PI(4,5)P_2_ from the PM to present this lipid to PLC-β3 and boost its activity (Zhong et al., 2019). Overall, it remains unclear why ORP4L has distinct localizations, why it can bind sterol, PI(4)P and PI(4,5)P_2_ and why it interacts with vimentin.

ORP9 exists in a long form, ORP9L, whose domain organization and subcellular localization resemble those of OSBP. It binds to VAP proteins and associates with the *trans*-Golgi/TGN via a PH domain (Wyles and Ridgway, 2004; Ngo and Ridgway, 2009). *In vitro*, ORP9L can sequester sterol or PI(4)P (Liu and Ridgway, 2014) and transfer sterol between synthetic membranes (Ngo and Ridgway, 2009; Liu and Ridgway, 2014). ORP9L appears to be important for maintaining ER-to-Golgi vesicular transport and Golgi organization, as well as sterol levels in the post-Golgi and endosomal compartment (Ngo and Ridgway, 2009). ORP9L impacts Golgi PI(4)P levels and cooperates with OSBP for building ER-Golgi contacts (Venditti et al., 2019). However, ORP9L seems functionally different from OSBP: its activity is decoupled from that of CERT and, presumably, ORP9L does not transfer sterol to the Golgi by sterol/PI(4)P exchange (Ngo and Ridgway, 2009). ORP9S is a shorter ORP9 variant that is absent from the Golgi surface as it lacks the PH domain. However, it can downregulate Golgi PI(4)P levels in a VAP-dependent manner (Liu and Ridgway, 2014), maybe by occupying preformed ER-Golgi contacts. ORP9S can disorganize Golgi structure and ER-to-Golgi trafficking (Ngo and Ridgway, 2009). Overall, it is unclear whether ORP9 variants are sterol/PI(4)P exchangers.

ORP10 is one of the two members of the ORP subfamily VI (Figure 5A). It was first described as a microtubule-associated protein that localizes to the Golgi complex and controls ER-Golgi trafficking to modulate the secretion of apolipoprotein B-100 from hepatocytes (Nissila et al., 2012). ORP10 associates with the Golgi complex via its PH domain (Nissila et al., 2012) and its ORD can trap PS (Maeda et al., 2013). Silencing ORP10 alters the PS content at the TGN, but also severely reduces ER-TGN contact sites (Venditti et al., 2019). ORP10 has no FFAT motif and heterodimerizes with ORP9 (Nissila et al., 2012) to colocalize with OSBP in ER-Golgi contacts, possibly for coordinating PS transfer, by PS/PI(4)P exchange, with ER-to-Golgi sterol transfer. These observations partially explain how PS accumulates at the TGN (Leventis and Grinstein, 2010).

## Discussion

Lipid exchangers belonging to three distinct families make critical connections between lipid metabolism, vesicular trafficking and signaling pathways, through the provision of lipids to organelles. Many of these ensure the directional transfer of lipids and are propelled by metabolic pathways that use CTP and ATP energy to create PI and/or PIPs. The biochemical features of lipid exchange modules might offer safeguard mechanisms to prevent any futile transfer of lipids between organelles. *In vitro* analyses of Osh4p revealed tight coupling between the rate of sterol and PI(4)P transfer that occur in opposite directions between membranes (Moser Von Filseck et al., 2015b). Simply stated, if sterol or PI(4)P is missing, the transfer of the other ligand is much slower. Likewise, mutations that abrogate the affinity of Osh4p for PI(4)P also shut down its sterol transfer capacity. Such a coupling might also exist for other ORP/Osh proteins. For instance, OSBP, Osh6p, or ORP2 fail to transfer sterol or PS in the cells when their ability to recognize PI(4)P is compromised (Mesmin et al., 2013; Moser Von Filseck et al., 2015a; Wang et al., 2019). Thus, one can assume that the ORP/Osh-mediated transfer of sterol and PS cannot occur between organelles if there is insufficient PIP available. Moreover, in the absence of transfer from the ER, feedback mechanisms can be activated to limit the accumulation of sterol or PS in that compartment (Kuge et al., 1998; Brown et al., 2018). It is unclear whether tight coupling exists between PA and PI transfer catalyzed by Class II PITP. This is perhaps less critical as these two lipids are metabolically connected: a lack of PA or PI synthesis can ultimately limit the production of the other lipid.

Interestingly, several lipid exchangers, as they share an analogous architecture, are regulated at contact sites by similar mechanisms. In complex LTPs such as OSBP, ORP5/8 and Nir2, an interdependence exists between the lipid transfer module and the Golgi/PM-targeting domain that both have the same lipid specificity (e.g., PI(4)P in the case of OSBP and PA in the case of Nir2/Nir3) (Mesmin et al., 2013; Kim et al., 2015). This adjusts the mobilization of these LTPs at contact sites as function of the amount of available lipid to be transported. Also, the existence of homologous LTPs (e.g., ORP5/8 and Nir2/3), with slightly different aptitudes to target PIPs or PA at the PM and to transfer these lipids, support exchange processes that occur with specific velocities and for precise lipid concentration thresholds (Chang and Liou, 2015; Sohn et al., 2018). This introduces subtle ways to regulate PIP metabolism at the PM in response to external stimuli. It is unclear why certain exchangers rely on VAP to associate with the ER whereas others are directly anchored to this compartment (Amarilio et al., 2005; Chung et al., 2015).

It is also worth noting that, despite being structurally unrelated (Chiapparino et al., 2016), the lipid transfer modules of Sec14p, PITPs and ORPs/Osh proteins share features that seem important for their activity. All of these have a conserved fingerprint or bar code to recognize PI or PIPs but have evolved to recognize diverse secondary ligands and fulfill specific functions. Moreover, in these modules, a lid/gate controls the access to the lipid-binding pocket and modifies how these modules bind to membrane when it closes. Indeed, these domains adopt a membrane-docking conformation when they are empty and a soluble state once they encapsulate a lipid. We recently reported how Osh6/7p can interact with the PM during an PS/PI(4)P exchange cycle and then escape from this membrane, which is highly anionic, to return to the ER, whose surface is more neutral (Yeung et al., 2008; Leventis and Grinstein, 2010). The avidity of Osh6p for anionic membranes is strongly reduced, once it extracts PS or PI(4)P, due to the closing of its lid that contains an anionic D/E-rich motif (Lipp et al., 2019). This helps Osh6p to self-limit its dwell time on membranes and thereby, to efficiently transfer lipids at ER-PM interface. Of note, ORP1 ORD was found to associate less with the membrane upon sequestering its ligand (Dong et al., 2019). Likewise, PITPα and PITPβ have less membrane-binding affinity when trapping PI (Panagabko et al., 2019). This might explain why they rapidly shuttle lipids between membranes *in vitro*, and possibly between organelles. Of note, chemical intervention and maybe endogenous phosphorylation can freeze PITPβ in an empty and membrane-docking conformation by impairing the extraction of the lipid and the closing of the gate (Shadan et al., 2008). Consequently, PITPβ remains tightly bound to the organelle surface. The PITD of Nir2 might have a dual role, i.e., to exchange lipids at ER-PM contacts in stimulated cells or to recruit Nir2 to the Golgi membrane in resting cells (Kim et al., 2013). A mechanism that is able to control the conformation of the PITP domain is most probably at play. α-TTP has less affinity for membranes in the presence of α-tocopherol (Morley et al., 2008) and recent data suggest that Sec14p dissociates from membrane when it extracts a ligand (Sugiura et al., 2019). Considered together, these data suggest that the lipid exchangers have mechanisms to dissociate from the membrane upon lipid extraction that offer kinetic advantages. Whether this is valid for most ORP/Osh proteins or Class II PITP awaits examination.

Our knowledge of the LTP modes of action directly relies on our ability to establish how they displace lipids *in vitro* or in cells. We succeeded to show that ORP/Osh proteins are lipid exchangers as we could measure their ability to simultaneously transfer lipids along opposite directions between membranes. Moreover, it was possible to analyze how these exchange processes rely on Sac1, the lipid content and curvature of membrane, and the acyl chain composition of ligands (Mesmin et al., 2013; Moser Von Filseck et al., 2015b). In cells, one can measure the exchange activity of ORP/Osh proteins and Class II PITP (Mesmin et al., 2013, 2017; Chang and Liou, 2015; Chung et al., 2015; Kim et al., 2015; Moser Von Filseck et al., 2015a; Sohn et al., 2018; Wang et al., 2019). This is feasible due to the nature of lipids under scrutiny. Indeed, sterol transfer activity can be measured *in vitro* and in cells using a natural and fluorescent sterol or genetically encoded-fluorescent biosensors. Likewise, natural PIPs or PS can be tracked between artificial or cellular membranes by fluorescent probes based on lipid-binding domains. In contrast, it has always been difficult to analyze *in vitro* how PC/PI exchangers function due to the identity of the ligands. First, as PC is a major background lipid of cell membranes, it is delicate to infer how this lipid, as a ligand, contributes to the speed of the PC/PI exchange process. Secondly, the technology does not yet exist to follow the transfer of natural PC and PI in real-time. One must use radiolabeled lipids in assays lacking temporal resolution or fluorescently labeled PC or PI with potential biases due to the fluorescent moieties. However, efforts have recently been made to overcome these difficulties using single-angle neutron scattering (Sugiura et al., 2019). It is also challenging to follow the cellular activity of PC/PI exchangers due to the lack of PC or PI biosensors. Fortunately, new tools have been designed to directly or indirectly measure the presence of PI in the membrane (Pemberton et al., 2020; Zewe et al., 2020). They revealed that PI is abundant in the Golgi membrane except if the production of PI is eliminated at the ER. This suggests that PI transfer occurs and creates a Golgi PI pool that is used thereafter to produce PI(4)P. In contrast, the PM contains hardly any PI, suggesting that PI is directly presented to PI 4-kinases. These tools might help to define whether Sec14p and PITPs move PI in the cell and how quickly, and whether they directly present PI to PI 4-kinases or deliver PI to the membrane. It will be also interesting to obtain structural and kinetic data on Class II PITPs, in particular Nir2, to accurately define how PA and PI are recognized and counter exchanged. The use of probes for PI and PA (Spo20) (Kim et al., 2015) might help to achieve this goal and to improve our understanding of how Class II PITPs efficiently respond to the consumption of PIPs by PLC-based signaling cascades.

## Author Contributions

N-FL, SI, JM, and GD conceptualized and wrote the manuscript. All authors contributed to the article and approved the submitted version.

## Conflict of Interest

The authors declare that the research was conducted in the absence of any commercial or financial relationships that could be construed as a potential conflict of interest.
